# Molecular Epidemiology, Phenotypic and Genomic Characterization of Multidrug-Resistant Enterococcus Faecium Isolated from Bovine Mastitis in Ningxia, China (2019–2024)

**DOI:** 10.3390/microorganisms14071424

**Published:** 2026-06-29

**Authors:** Yarui Qiao, Xinyuan Zhang, Ruixin Jing, Jun Du, Yang Liu, Yonglin Zhou, Dongtao Zhang, Xuezhang Zhou

**Affiliations:** 1 College of Life Sciences, Ningxia University, Yinchuan 750021, China; 15209665024@163.com (Y.Q.);; 2 Key Laboratory of Conservation and Utilization of Biological Resources with Western Characteristics, Ministry of Education, Yinchuan 750021, China

**Keywords:** *E. faecium*, resistance, molecular epidemiology, genomic analysis

## Abstract

Multidrug-resistant (MDR) *Enterococcus faecium* is an opportunistic pathogen. Its resistance and virulence genes can spread through the food chain, posing risks to public health. This study investigated the antimicrobial resistance and genomic characteristics of MDR E. faecium isolated from milk samples from cows with mastitis in Ningxia between 2019 and 2024. From 2019 to 2024, 1341 milk samples were collected in Yinchuan, Yinnan, and Yinbei. MDR *E. faecium* was identified using plate screening, mass spectrometry, broth microdilution, and hemolysis detection. Whole-genome sequencing enabled SNP, MLST, pan-genome, and COG analyses, focusing on ARGs and MGEs. MRPP, AMOVA and PCoA were applied to compare gene communities and identify driver genes. Ninety-one *E. faecium* strains were isolated. Resistance to florfenicol, ceftiofur, and chloramphenicol exceeded 60%, while resistance to vancomycin and linezolid showed an overall increasing trend over the study period. Phylogenetic clustering revealed two subtypes, three clades, and 10 novel STs. Spearman correlation analysis revealed strong positive correlations among the resistance genes *optrA*, *cfr(A)*, and *vanF*. Antibiotic resistance, particularly MDR, increased over time, and strains carried diverse ARGs and MGEs. Overall, strengthened surveillance of mastitis-derived *E. faecium* is warranted to support the control of bovine mastitis and safeguard public health.

## 1. Introduction

Multidrug-resistant (MDR) *Enterococcus* constitutes a clinically significant genus characterized by strong opportunistic pathogenicity and a pronounced ability to disseminate resistance determinants [1]. *Enterococcus* species are firmly established as major agents of nosocomial infections, including endocarditis, urinary tract infections, and bloodstream infections [2,3]. Of particular concern is the increasing resistance to last-line agents such as vancomycin and linezolid, which are critical for treating severe enterococcal infections in humans. The resistance profile of *E. faecium* encompasses both intrinsic and acquired mechanisms that confer resistance to clinically important antimicrobials [4]. Mobile genetic elements (MGEs), including conjugative plasmids, transposons, and insertion sequences, serve as efficient vehicles for resistance gene transfer. Plasmid pRE25, which has been detected in both *E. faecalis* and *E. faecium* [5], can concurrently harbor *tetM, ermB,* and *cat* [6], allowing a single conjugation event to generate a multidrug-resistant phenotype. The pathogenicity of enterococci is closely linked to their antimicrobial resistance. The genome of *E. faecium* generally lacks a fully operational restriction-modification (R-M) system [7], providing a permissive genomic milieu for the uptake of foreign virulence and resistance loci [8]. This deficiency, compounded by an abundance of MGEs [9], renders *E. faecium* a highly adaptable pathogen capable of rapidly accruing and spreading resistance traits under sustained antibiotic selection pressure.

Within the One Health framework, enterococci serve as important indicator bacteria connecting food, the environment, animals, and humans, and their resistance and virulence risks have emerged as a public health research priority [10,11]. Consequently, the emergence of enterococci as veterinary pathogens is drawing increasing scrutiny. In dairy herds, *Enterococcus* has been associated with bovine mastitis, an economically damaging disease that undermines both milk quality and animal welfare [12]. Mastitis-associated *E. faecium* frequently carries resistance to multiple first-line antibiotics, substantially narrowing therapeutic choices and intensifying concern over the environmental circulation of resistance genes within livestock operations [13,14,15]. Transmission among cattle occurs through direct animal contact and contaminated milking apparatus, facilitating both the persistence of mastitis within herds and the expansion of a regional resistome [16]. Affected cows may develop clinical or subclinical mastitis, shedding the organism into milkv [17]. Horizontal transfer of resistance genes from *E. faecium* to commensal or pathogenic bacteria inhabiting the udder microenvironment further compounds the issue, transforming a localized infection into a broader reservoir of transmissible resistance determinants [18,19]. This interplay fosters a self-perpetuating cycle in which imprudent antibiotic use in husbandry and suboptimal milking hygiene accelerate the dissemination of resistance, culminating in therapeutic failure and mounting disease control burdens [20,21].

Although antimicrobial resistance of *E. faecium* in bovine mastitis has been reported previously [22], systematic investigations focusing on the genomic characteristics of longitudinal samples from the Ningxia region between 2019 and 2024 remain lacking [23]. This study aimed to recover and characterize *E. faecium* strains acting as causative agents in mastitic milk, rather than merely estimating the prevalence of enterococcal mastitis. To this end, this study, for the first time, performed whole-genome sequencing and comparative genomic analysis on MDR *E. faecium* isolates recovered from bovine mastitis over six consecutive years in Ningxia, thereby filling the data gap regarding the temporal evolution of antimicrobial resistance and molecular epidemiology in this region. The findings provide a scientific basis for the risk management of MDR-*E. faecium* mastitis and the improvement of dairy safety controls.

## 2. Materials and Methods

### 2.1. Collection and Identification of Isolates

Between 2019 and 2024, a total of 1341 milk samples were collected from Holstein cows with clinical mastitis at dairy farms in Yinchuan, Yinnan, and Yinbei, Ningxia. Clinical mastitis was defined as the presence of grossly abnormal milk (e.g., clots, flakes, or watery appearance), udder swelling, or heat, with or without systemic signs. Subclinical mastitis (normal milk appearance but elevated somatic cell count) was excluded. After disinfecting the udder with 75% alcohol and discarding the first three streams, approximately 15 mL of milk was collected into sterile tubes, stored at 4 °C, and transported to the laboratory within 6 h. In the laboratory, milk samples were streaked onto blood agar plates and incubated aerobically. Predominant colonies were picked to obtain pure cultures of clinically relevant bacteria. Predominant colonies are defined as single colonies that account for more than 50% of the total colony count on the plate and exhibit consistent morphology (identical size, color, and hemolytic pattern). If two or more distinct colony morphotypes were both numerically dominant, three single colonies of each morphotype were picked for further identification. Subsequently, these isolates were inoculated onto Pfizer *Enterococcus* selective agar and *Enterococcus* chromogenic medium, and incubated under aerobic conditions at 37 °C for 24 to 48 h to screen for *Enterococcus* spp. *Enterococcal* isolates were confirmed as *E. faecium* by MALDI-TOF mass spectrometry (Bruker Daltonik, Bremen, Germany) with a score ≥ 2.0 for species-level confidence, and subsequently tested for hemolysis on 5% sheep blood agar. All confirmed *E. faecium* strains were stored at −80 °C in brain heart infusion broth with 15% glycerol. Notably, *E. faecium* is an opportunistic mastitis pathogen and not the primary cause of most mastitis cases; this study aimed to recover and characterize *E. faecium* strains acting as causative agents in mastitic milk, not to estimate the prevalence of enterococcal mastitis.

### 2.2. Detection of Phenotypic Antibiotic Resistance

In this study, the minimum inhibitory concentration (MIC) of antibiotics was determined to evaluate the susceptibility of isolated strains. *Enterococcus faecalis* ATCC-29212 served as the quality control strain. The testing method and breakpoints followed the broth microdilution protocol recommended by the Clinical and Laboratory Standards Institute (CLSI, Wayne, PA, USA), according to the CLSI M100-Ed33 (2023) standard. MIC values were determined using the BD Phoenix^™^ 100 Automated Microbiology System (Sparks, MD, USA) for bacterial identification and susceptibility testing. The 14 antibiotics tested were lincomycin, tetracycline, ciprofloxacin, vancomycin, fosfomycin, ceftiofur, ampicillin, chloramphenicol, gentamicin, linezolid, trimethoprim, florfenicol, doxycycline, and erythromycin. To ensure accurate MIC determination for all isolates (including resistant strains), the two-fold serial dilution ranges were deliberately extended beyond the standard CLSI ranges, covering a broad spectrum from 0.125 µg/mL to 1024 µg/mL and encompassing all relevant CLSI breakpoints. Each test was performed in triplicate using independent assays on three different days.

### 2.3. Whole-Genome Sequencing and Assembly

Whole-genome sequencing was performed by Sangon Biotech (Shanghai) Co., Ltd. (Shanghai, China), using the Illumina NovaSeq 6000 platform (San Diego, CA, USA) with a paired-end 150 bp read length. The average sequencing depth was approximately 450× (range: 300–1600×, with a few samples exceeding 2900×). For raw sequencing data, statistical evaluation and quality assessment were conducted using Fastp version 0.19.6. Quality control of raw reads was performed under the following criteria: (1) removing reads with low-quality bases (quality value ≤ 20) exceeding 40%; (2) removing reads with N bases (unknown bases) above 10%; and (3) eliminating reads overlapping ≥15 bp with adapter sequences and with <3 mismatches. Illumina sequencing data were assembled using SPAdes (http://bioinf.spbau.ru/spades, accessed on 12 March 2025). GapFiller (https://sourceforge.net/projects/gapfiller/, accessed on 20 March 2025) was applied to fill gaps in contigs, and Pilon (https://github.com/broadinstitute/pilon, accessed on 25 March 2025) was used for sequence correction to resolve base call errors and small insertions/deletions during assembly. To filter homopolymer contamination, reads with sequencing depth less than 0.35 times the average depth were removed. This threshold was adopted based on the SPAdes assembler’s default cov-cutoff parameter and previous studies [24,25]. The final assembly results were then obtained.

### 2.4. Phylogenetic and Pan-Genome Analyses

Pan-genome analysis of the isolates was performed with Roary v3.13.0 (https://github.com/sanger-pathogens/Roary, accessed on 3 April 2025) using an 85% sequence identity threshold (-i 85) to define homologous gene clusters and to generate datasets of the core genome (genes shared by all strains), accessory genome (genes present in a subset of strains), and strain-specific genes [26]. Single-nucleotide polymorphisms (SNPs) were extracted from the core genome alignment using SNP-sites v2.5.1 (https://github.com/sanger-pathogens/snp-sites, accessed on 5 April 2025) to generate a core SNP matrix for phylogenetic inference [25]. Maximum likelihood (ML) phylogenetic trees were constructed with RAxML under the GTR + GAMMA model, and branch support was evaluated with 1000 bootstrap replicates; branches with bootstrap values ≥70% were considered robust. To assign evolutionary lineages, 67 publicly available *E. faecium* genomes were retrieved from NCBI (https://www.ncbi.nlm.nih.gov/, accessed on 10 April 2025), selected to cover diverse sources and geographic regions, and classified into clades A1, A2, and B according to Lebreton [27]. Strain details are provided in Supplementary Table S8. Protein functions were annotated by aligning predicted protein sequences against the Clusters of Orthologous Groups (COG) database using Diamond, with functional categories and putative biological roles assigned based on the resulting best matches.

### 2.5. Bioinformatics Analysis

The sequence types (STs) of strains were identified using multilocus sequence typing (MLST) v2.23.0 software. Novel STs identified in this study were submitted to the PubMLST *E. faecium* database (https://pubmlst.org/organisms/enterococcus-faecium, accessed on 15 April 2025) for assignment of official allele numbers and ST designations. ResFinder v4.5.0 software was applied for annotation of antimicrobial resistance genes (ARGs), with a minimum sequence identity set at 90% and minimum coverage at 80%. The ABRicate tool, combined with the virulence factors database (VFDB), was used to detect virulence genes showing ≥90% sequence identity with database entries. For plasmid replicon typing, PlasmidFinder v2.1.6 software was employed to align genomic sequences against the plasmid replicon database with default parameters (minimum identity 95% and minimum coverage 80%). ISEScan v1.7.2.3 software was utilized to scan genomic sequences with a hidden Markov model and identify insertion sequence (IS) elements.

### 2.6. Statistical Analysis

The analytical methods, multi-response permutation procedure (MRPP), analysis of molecular variance (AMOVA) and principal coordinates analysis (PCoA), were employed to examine the significance of community differences and driving factors of ARGs and virulence genes in *E. faecium* from Ningxia. Results of these analyses (MLST, ARGs, virulence genes, plasmids, and IS distribution) were correlated and integrated with strain sampling information, including time and location. Visualization was carried out using the ggplot2 package (https://ggplot2.tidyverse.org, accessed on 20 April 2025) in R. Phylogenetic trees were generated on the online platform Interactive Tree of Life (iTOL, https://itol.embl.de/, accessed on 20 April 2025). The co-occurrence network of ARGs was constructed using Cytoscape v3.8.0.

## 3. Results

### 3.1. Sources of Milk Isolated E. faecium

A total of 1341 milk samples were collected, yielding 91 *E. faecium* strains, with only one strain retained per sample for subsequent analysis (if multiple colonies from the same sample were identified as *E. faecium,* only one was selected to avoid bias from duplicate sequence homology). The isolation time, geographical location, and hemolytic properties were documented (Table S1). The isolation rate of *E. faecium* was 6.8%. These isolates originated from three regions of the Ningxia Hui Autonomous Region: Yinchuan (*n* = 30), Yinnan (*n* = 45), and Yinbei (n = 16). The collection period spanned 2019–2024. The annual isolates were: 2019 (*n* = 16), 2020 (*n* = 15), 2021 (*n* = 15), 2022 (*n* = 14), 2023 (*n* = 15), and 2024 (*n* = 16). Hemolysis testing was carried out on isolated strains. The predominant type was β-hemolysis (*n* = 56), followed by α-hemolysis (*n* = 21) and γ-hemolysis (*n* = 14). The detection rate of *E. faecium* varied significantly across years, ranging from 4.2% to 16.3%. The highest detection rate occurred in 2022 (16.3%), which was 3.9 times higher than the rate in 2023 (4.2%). The difference in detection rates among years was statistically significant (Pearson’s chi-square test:χ^2^ ≈ 34.56, df = 5, *p* < 0.001). However, the differences in detection rates among regions did not reach statistical significance (Pearson’s chi-square test: χ^2^ ≈ 5.86, df = 2, *p* = 0.053) (Table 1).

### 3.2. MDR Analysis of Isolated Strains

To evaluate the antibiotic resistance of isolated strains, the broth microdilution method was used to test susceptibility against 14 antibiotics (Table S2). According to the Clinical and Laboratory Standards Institute [28] and the European Committee on Antimicrobial Susceptibility Testing [29], *Enterococcus* spp. exhibit predictable intrinsic insensitivity (i.e., naturally occurring resistance) to lincosamides (e.g., lincomycin, clindamycin), cephalosporins (e.g., cefepime, ceftiofur), and sulfonamides (e.g., sulfadiazine). Consequently, in vitro susceptibility testing results for these agents are generally not recommended for guiding clinical therapy. Nevertheless, to provide a complete dataset, the MIC results for these four drugs are reported in this study. All isolates were resistant to lincomycin and tetracycline (100%), and thus these results are not further displayed in the figure. For detailed data, please refer to Supplementary Table S2.

**Figure 1:**
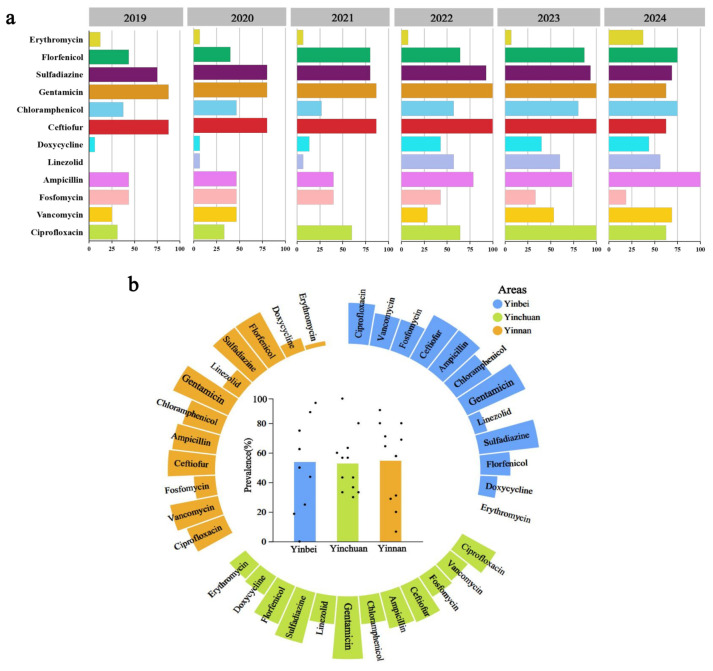
Prevalence of antibiotic resistance phenotypes among 91 isolates. (**a**) Temporal changes in resistance from 2019 to 2024. Horizontal bars show percentages of isolates resistant to each drug class. (**b**) Circular bar plot showing resistance by region. Central bars represent average proportions, scatter points indicate variations, and the outer ring shows resistance to individual antibiotics.

Resistance rates to florfenicol (64.84%), ampicillin (63.74%), ciprofloxacin (58.24%), and chloramphenicol (53.85%) exceeded 50%. Notably, the resistance rates of vancomycin and linezolid—last-line antibiotics in human medicine reached as high as 37.36% and 30.77%, respectively, among the bovine mastitis *E. faecium* isolates in this study. Further analysis revealed that resistance to both vancomycin and linezolid emerged predominantly during 2022–2024, suggesting a rapid trend of dissemination. Resistance from 2022 to 2024 was generally higher than from 2019 to 2021. With time, resistance to antibiotics, including florfenicol, doxycycline, and ceftiofur sodium, gradually increased (Figure 1a). No significant difference in resistance was observed among regions. However, the number of resistant antibiotic types followed the order: Yinchuan > Yinnan > Yinbei (Figure 1b).

### 3.3. Phylogenetic and Pangenomic Analyses of Isolated Strains

#### 3.3.1. Pangenomic Analysis of Isolated Strains

**Figure 2:**
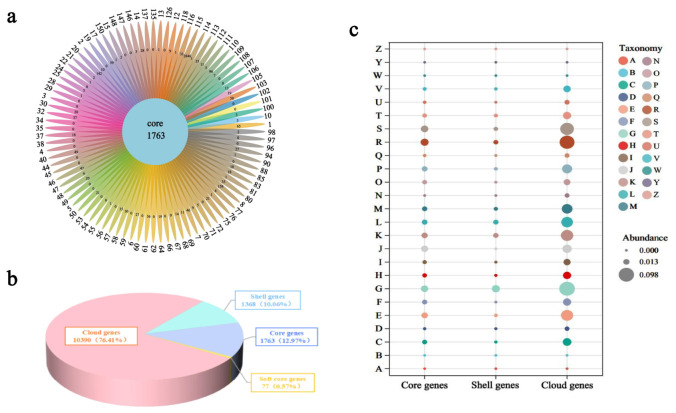
Genomic analysis of milk-derived *E. faecium*. (**a**) Petal map of core genes of isolated strains. (**b**) Ratio of various genes in isolated strains. (Percentages are rounded to one decimal place; therefore, the sum may not be exactly 100%.) (**c**) Abundance distribution of taxonomic units by pan-genome categories (core, shell, and cloud). Points are colored by COG functional category (A–W, Y, and Z), and point size represents the number of genes assigned to each category, illustrating the distribution of genes across functional classes. The letter codes represent: A, RNA processing and modification; B, chromatin structure and dynamics; C, energy production and conversion; D, cell cycle control, cell division, and chromosome partitioning; E, amino acid transport and metabolism; F, nucleotide transport and metabolism; G, carbohydrate transport and metabolism; H, coenzyme transport and metabolism; I, lipid transport and metabolism; J, translation, ribosomal structure, and biogenesis; K, transcription; L, replication, recombination, and repair; M, cell wall/membrane/envelope biogenesis; N, cell motility; O, posttranslational modification, protein turnover, and chaperones; P, inorganic ion transport and metabolism; Q, secondary metabolite biosynthesis, transport, and catabolism; R, general function prediction only; S, function unknown; T, signal transduction mechanisms; U, intracellular trafficking, secretion, and vesicular transport; V, defense mechanisms; W, extracellular structures; Y, nuclear structure; and Z, cytoskeleton, based on the COG database.

According to Roary default thresholds, core genes are defined as those present in ≥99% of isolates, soft core genes in 95–99%, shell genes in 15–95%, and cloud genes in <15%. Pangenomic analysis showed that the 91 isolates shared 1763 core genes, representing 12.97% of pan-genome genes (13,598 genes) (Figure 2a). Shell genes accounted for 10.06% (1368 genes), while cloud genes accounted for 76.41% (10,390 genes) (Figure 2b). This indicates that the 91 *E. faecium* isolates in this study possess extremely high genomic diversity. This finding is consistent with the characteristic of *E. faecium* as a highly recombinogenic species, reflecting that the bacterium has acquired a large number of variable accessory genes through frequent horizontal gene transfer during its long-term evolutionary process [30,31]. To assess the openness of the pan-genome, we performed accumulation analysis with random subsampling (100 replicates for each genome count level). As shown in the pan-genome accumulation curve (Supplementary Figure S1), the number of pan-genes continued to increase substantially with the addition of more genomes, from 1 to 91, without reaching an observable plateau. In contrast, the core genome declined rapidly and stabilized after approximately 20 genomes.

Clusters of orthologous group (COG) analysis indicated that genes related to carbohydrate transport and metabolism (G) were most abundant, followed by transcription (K) and replication, recombination, and repair (L). In contrast, genes for intracellular trafficking, secretion, and vesicular transport (Q) and those for biosynthesis, transport, and catabolism of secondary metabolites (U) were markedly fewer. Genes of unknown function (S) accounted for a relatively high proportion (Figure 2c). The sequencing data has been uploaded to NCBI (BioProject ID: PRJNA1344258).

#### 3.3.2. Phylogenetic Analyses

**Figure 3:**
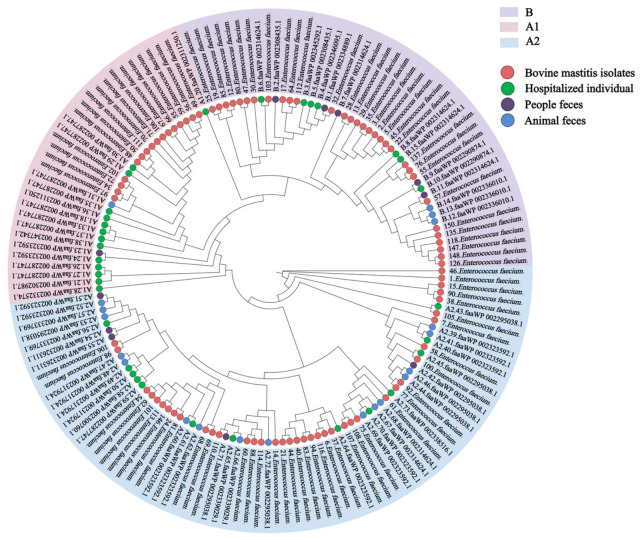
Maximum likelihood phylogenetic tree based on whole-genome SNPs. The tree was built from recombination-filtered whole-genome SNP (wgSNP) alignments of 158 isolates using the GTR + F + ASC + G4 model. The clades are color-coded: clade B is in dark blue, clade A1 is in red, and clade A2 is in gray.

**Figure 4:**
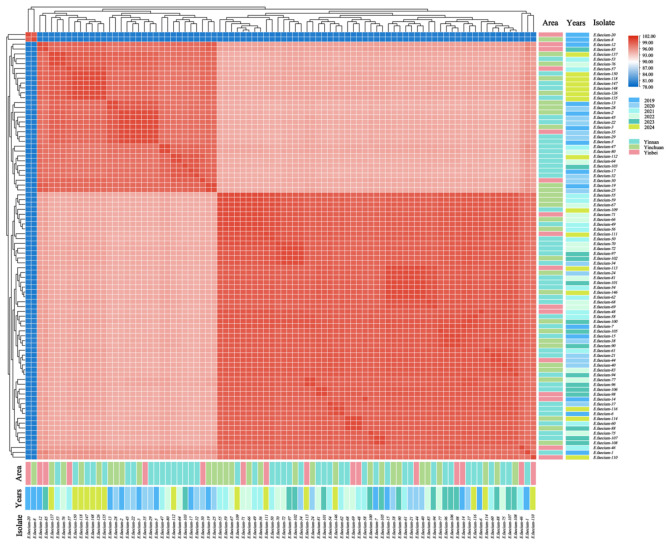
ANI analysis of *E. faecium* isolates. Heatmap color intensity indicates ANI values between strain pairs: warmer colors (red) reflect higher ANI (greater similarity), while cooler colors reflect lower ANI (greater divergence). Dendrograms show hierarchical clustering, and the right panel lists isolation time, location, and strain names.

An SNP-based phylogenetic tree was constructed from 807 shared single-copy orthologous genes. This tree compared the isolated strains with those reported in Lebreton’s study and classified them into three evolutionary branches: A1, A2, and B (Figure 3). Strains were divided into two genetic subtypes according to branch clustering; intra-subgroup gene identity was high. ANI analyses revealed that the strains predominantly formed a closely related genetic cluster, with only a few strains branching off independently. No clear temporal structuring was detected, and strains from all three regions were broadly intermingled without forming geographically restricted clades (Figure 4).

#### 3.3.3. MLST Analyses

MLST analysis identified 10 known sequence types (STs) and 10 novel STs among the 91 milk-derived *E. faecium* isolates (Table 2). The most prevalent known ST was ST18 (8/91, 8.8%), predominantly isolated from Yinchuan and persisting across multiple years, suggesting endemic circulation of this clone in local dairy herds. ST27 (7/91, 7.7%) and ST361 (6/91, 6.6%) were both primarily recovered from Yinnan in 2024, indicating possible recent introduction or local expansion. Other known STs included ST80 (*n* = 4), ST296 (*n* = 3), ST771 (*n* = 3), and ST17, ST224, ST956, and ST1532 (*n* = 2 each). The remaining 31 isolates (34.1%) were assigned to 10 novel STs, among which ST3088 (*n* = 3), ST3086, ST3089, ST3094, and ST3097 (*n* = 2 each) were the most frequently represented. All novel STs were submitted to the PubMLST database (https://pubmlst.org/organisms/enterococcus-faecium, accessed on 3 April 2026) for official allele assignment and were assigned new ST designations (submission ID: BIGSdb-20260403155110-2937026-78255).

### 3.4. Genomic Characteristics of Isolated Strains

#### 3.4.1. Resistance Gene Analysis

**Figure 5:**
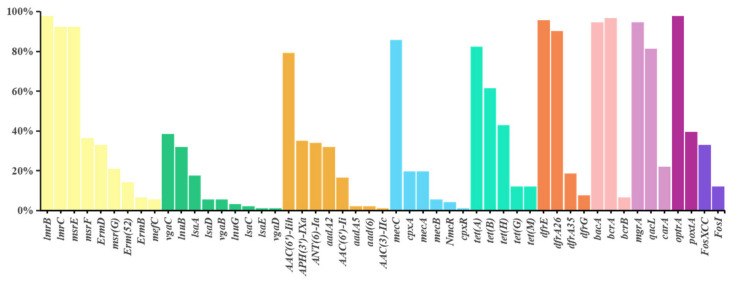
Carrier rates of ARGs in dairy-derived *E. faecium*. The bar chart shows proportions of genomes carrying specific ARGs, with colors representing antimicrobial classes. Only high-prevalence resistance genes are displayed.

A large number of drug resistance genes were detected in *E. faecium* isolates. Among these ARGs, multidrug resistance genes showed the highest detection rate (49.8%), followed by glycopeptide resistance genes (18.9%) and tetracycline resistance genes (8.3%). Specifically, the polymyxin resistance gene *msbA* was present in all isolates. The detection rates of *TaeA, adeR*, *arlS*, *efrA*, and *macB* reached 98.9% (90/91). Additionally, detection rates of the vancomycin resistance gene *vanF*, the florfenicol resistance gene *optrA*, the tetracycline resistance gene *otr(B)*, and the sulfonamide resistance gene *dfrE* all exceeded 95% (Figure 5).

#### 3.4.2. MGEs Analysis

**Figure 6:**
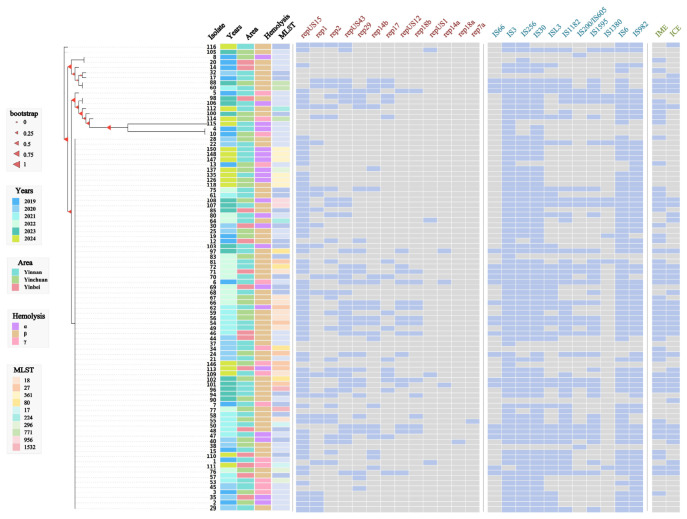
Distribution of MGEs and conjugative transfer elements. On the left, a phylogenetic tree of isolates was constructed from 16S rRNA sequences. Triangle markers and their colors on branches correspond to bootstrap values, where darker colors and larger markers indicate higher confidence. Annotation columns next to the tree show isolation year, geographic origin, hemolytic phenotype, and MLST of each strain. The heatmap on the right uses blue tiles to indicate presence (blue) or absence (gray) of conjugative transfer elements, including plasmid replication genes, IS elements, and IME or ICE integrative elements.

MGEs were analyzed in 91 isolates (Figure 6). The isolates carried 13 plasmid replicon types in total. Seventy-seven isolates (84.6%, 77/91) carried at least one plasmid, and 37 of them (48.1%, 37/77) contained more than four plasmids. Carriage rates of repUS15, repUS43 and rep2 were 87.0% (67/77), 44.2% (34/77), and 37.7% (29/77), respectively. Eighty-eight isolates (96.7%) harbored one or more insertion sequence (IS). The IS3 element had the highest rate (95.5%), followed by IS30 (89.8%), IS256 (86.4%), and IS6 (84.1%). Conjugative transfer elements were detected in 58 isolates. The main detected elements were integrative mobile elements (IME) and integrative conjugative elements (ICE), associated with the Type IV secretion system (T4SS), with detection rates of 88.0% (51/58) and 63.8% (37/58), respectively. Twenty-nine of these 58 isolates (50.0%, 29/58) carried resistance genes on conjugative transfer elements, mainly *tetM*, *tetL*, and *aac(6′)-Ii* (Table S3). Analysis of chromosomal mutation sites focused on *gyrA*, *parC*, and *pbp5*, with mutation rates of 19.8%, 19.8%, and 74.7% (Table S4).

### 3.5. Co-Occurrence and Correlation Analyses

#### 3.5.1. Co-Occurrence of Antibiotic Resistance Genes

**Figure 7:**
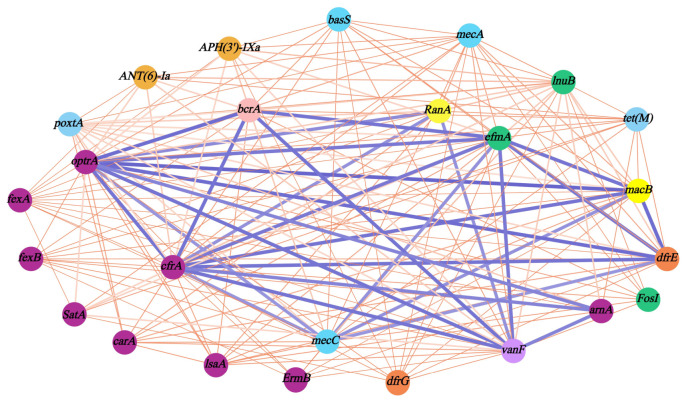
Co-occurrence network of ARGs within isolates, based on pairwise co-occurrence frequency across 91 genomes. The thickness and color intensity of connecting lines represent the co-occurrence frequency of genes in the same genome. Only high-prevalence resistance genes are shown.

Correlation analysis of genes mediating linezolid and vancomycin resistance revealed strong correlations among 10 resistance genes, including *optrA*, *cfr(A)*, *mecC*, and *vanF* (Figure 7). Notably, the *mecC* gene is a methicillin resistance gene typically closely associated with bovine-origin *Staphylococcus* aureus and a few other staphylococcal species [32].

#### 3.5.2. ST, Time, Region and ARG Associations

**Figure 8:**
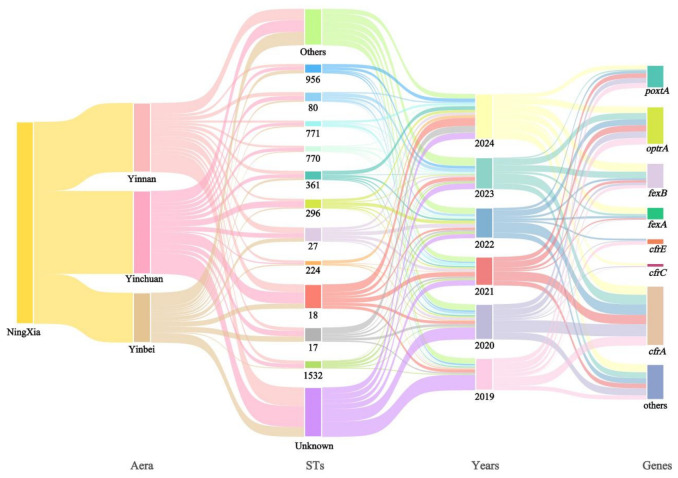
Sankey diagram showing ARG distribution across time points, regions, and MLST types.

A Sankey diagram analysis illustrated linezolid resistance-related genes across regions and ST subtypes from 2019 to 2024. Strains collected between 2023 and 2024 exhibited higher proportions of *poxtA* carriage compared with 2019–2022 (Table S1). A higher proportion of *fexA*-carrying strains were from 2024, while *fexB*-carrying strains were predominantly concentrated in 2023–2024. Unknown sequence types (unknown STs) accounted for a significantly higher proportion of strains from 2019 to 2020 than in subsequent years, suggesting greater genetic diversity among early isolates. The two major resistance genes, *optrA* and *cfr(A),* maintained consistently high detection rates throughout the period from 2019 to 2024 (Figure 8). ST770 has been identified as one of the predominant sequence types in clinical isolates and belongs to the CC17 clonal complex, a lineage closely associated with hospital infections and vancomycin resistance [33]. In this study, both ST770 and ST771 carried the *optrA* resistance gene. ST771, initially described as a novel sequence type, has been confirmed to exhibit strong biofilm-forming ability and has been isolated from *Cronobacter sakazakii* and *Cronobacter muytjensii* species [34].

### 3.6. Spatiotemporal Distribution of ARGs and VFs in Isolated Strains

To clarify the distribution of ARGs based on the relative abundance of resistance genes, MRPP and AMOVA were used to analyze community differences in isolates across regions and time periods (Table 3). The results showed significant differences in resistance genes among years. Specifically, MRPP and AMOVA analysis of adjacent year pairs revealed that most adjacent years had no significant differences in ARG community structure (*p* > 0.05), but there were significant shifts at key time nodes, with the most pronounced significant differences occurring between 2020 and 2021 (*p* = 0.012) and between 2023 and 2024 (*p* = 0.001). Additionally, the most obvious differences among non-adjacent year pairs were observed between 2019 and 2021 (*p =* 0.002) and between 2019 and 2022 (*p* = 0.003). In contrast, no significant regional differences were observed (*p* = 0.919).

**Figure 9:**
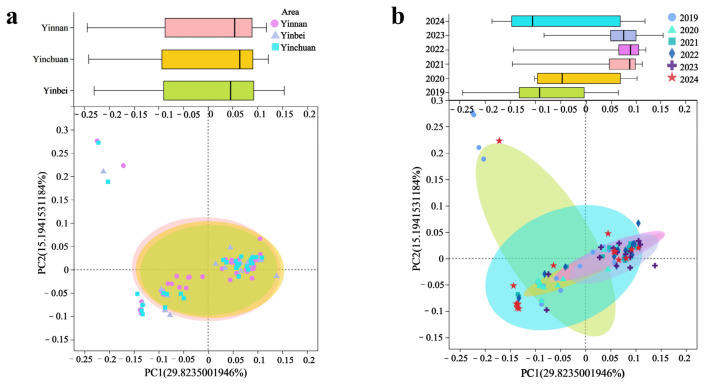
PCoA of ARGs categorized by time and region. (**a**,**b**) show the distribution and clustering characteristics of ARGs across different sampling regions and years.

PCoA was performed, and the results are shown in Figure 9: The Distribution of ARG types in isolates from Yinnan was broader. Genetic distributions of isolates from Yinnan, Yinbei, and Yinchuan overlapped. Distributions were scattered in 2019 and 2024 but concentrated between 2020 and 2023. Overall, ARG distribution patterns were similar across time and regions.

## 4. Discussion

The high prevalence of antimicrobial resistance (AMR) in mastitis-derived *E. faecium* is not an intrinsic trait but largely a consequence of the selective pressure exerted by antimicrobial usage in dairy farming, particularly for mastitis management [35]. In this study, 1341 mastitic milk samples were collected from distinct regions of Ningxia between 2019 and 2024. The overall isolation rate of *E. faecium* was 6.9% (91/1341), which is higher than the 4.9% previously reported by our group [19] and also exceeds the prevalence of *Enterococcus* spp. in clinical mastitis reported in recent international studies, including 4.86% in a large Italian multicenter survey [36] and 5.6% in a German nationwide investigation [37]. Taken together, these findings indicate that *Enterococcus* species, particularly *E. faecium*, are increasingly recognized as significant mastitis pathogens in dairy cows, warranting enhanced surveillance and further investigation into their transmission dynamics. Vancomycin and linezolid remain the last-resort antimicrobials for treating multidrug-resistant Gram-positive infections in clinical practice [38]. Alarmingly, we observed a progressive increase in vancomycin and linezolid resistance among mastitis-derived *E. faecium* isolates, directly challenging the therapeutic reliance on these agents.

MGEs, including plasmids, transposons, and integrons, are key mediators of horizontal resistance gene transfer in bacteria [39]. Plasmids carrying resistance genes demonstrate high interstrain transfer frequencies, with some high-mobility plasmids enabling rapid acquisition of resistance phenotypes by recipient bacteria [40]. In this study, 13 distinct plasmid replicon types were detected, and 59 isolates (67.0%) harbored 20 or more transposons. IS6 and IS256 were the most abundant. Notably, IS6 family insertion sequences promote interplasmid spread of the *optrA* resistance gene [41], whereas IS256 is strongly associated with ciprofloxacin and vancomycin resistance in *Staphylococcus aureus* [32]. Although the association between IS256 and resistance has been well documented in *S. aureus*, this evidence remains indirect for *E. faecium*, and further functional studies are needed to validate whether IS256 plays a similar role in this species. Conjugative transfer elements detected in the isolates included integrative mobile elements (IMEs) and integrative conjugative elements (ICEs) associated with the T4SS. The main resistance genes carried by these elements were *tetM*, *tetL* and *aac(6′)-Ii*. Chromosomal mutation analysis identified *pbp5* as the most frequently mutated locus, a genetic alteration strongly linked with ampicillin resistance [42].

Pangenomic analysis showed that the 91 *E. faecium* isolates exhibited a relatively low core genome proportion, sharing only 1763 core genes and accounting for merely 12.97% of the total pan-genome (shell genes: 10.06%; cloud genes: 76.41%). A low core genome proportion is indicative of exceptionally high genomic diversity, which is consistent with *E. faecium* being a highly recombinogenic species that frequently acquires accessory genes via horizontal gene transfer, indicating frequent gene flow within the regional *E. faecium* population. The pan-genome accumulation curve (Supplementary Figure S1) showed that the pan-genome size continued to increase substantially without reaching a plateau, confirming an open pan-genome. An open pan-genome means that new gene families, particularly those related to antimicrobial resistance, virulence, and metabolic adaptation, will likely be discovered as more *E. faecium* strains are sequenced [43]. This poses a considerable challenge for the control of *E. faecium*-associated infections and for public health surveillance. Our findings underscore the need for ongoing genomic surveillance of *E. faecium* in the context of One Health, especially in livestock-associated niches such as bovine mastitis.

Genomic screening revealed a broad repertoire of resistance genes. Notably, the vancomycin resistance gene *vanF* and the linezolid resistance gene *optrA* were detected in over 95% of all isolates, and strong associations were observed among *optrA*, *cfr(A)*, *mecC* and *vanF* [44,45]. The *mecC* gene is a methicillin resistance determinant typically associated with bovine-origin *Staphylococcus aureus* and a few other *Staphylococcal* species [32]. The occurrence of *mecC* in *E. faecium* in this study most likely originated from intergeneric horizontal gene transfer with bovine MRSA within the bovine mammary gland microenvironment [46]. This finding further supports the central role of enterococci as a “resistome sink” in cross-species transmission. The co-occurrence of these last-line resistance genes suggests their potential co-localization on shared MGEs. Previous studies have shown that *optrA* and *cfr(A)* can be co-carried by the same or co-transferable plasmids, often flanked by IS1216 [47,48]. IS1216 also drives intergeneric gene exchange between *Enterococcus* and *Staphylococcus* and may mobilize *mecC* [49,50]. Although we did not experimentally characterize the genetic contexts in our isolates, the available literature indicates that co-carriage of these genes likely results from their co-localization on shared or co-transferable MGEs.

The current *E. faecium* MLST database is predominantly populated with isolates of human origin (mainly from clinical nosocomial infections) and a limited number of animal-derived strains (e.g., from swine and poultry) [51]; the genetic background of milk-derived, particularly mastitis-derived, isolates is substantially underrepresented [52]. In this study, 10 novel sequence types were identified among the 91 isolates. These novel STs may represent endemic clones specific to the Ningxia region and have been formally deposited in the PubMLST database. A comparable pattern of novel ST discovery in livestock has been reported in other geographical settings [53], underscoring the global sampling bias in current MLST schemes. Sankey diagram analysis further demonstrated temporal shifts in ARG-ST associations: isolates collected during 2023–2024 exhibited elevated *poxtA* carriage relative to earlier years, and genotypes ST770 and ST771 were associated with *optrA*. Notably, ST770 belongs to the CC17 clonal complex, a lineage closely associated with hospital infections and vancomycin resistance [54]. The presence of this clinically relevant lineage in bovine mastitis isolates raises the possibility of cross-sector transmission [55], although the direction and route remain to be determined.

To systematically characterize the spatiotemporal dynamics and principal drivers of ARG composition in mastitis-derived *E. faecium* from Ningxia, we employed MRPP, AMOVA, and PCoA. MRPP and AMOVA consistently identified significant interannual variation in ARG community structure, with the most pronounced compositional shifts occurring between 2019 and 2021 and between 2019 and 2022. In contrast, spatial effects were not statistically significant, indicating that temporal, rather than geographic, factors predominantly shaped ARG evolution in these isolates. This finding highlights the importance of continuous longitudinal surveillance to capture the dynamic evolution of resistance gene reservoirs. Based on the findings of this study, we propose the following recommendations for veterinary practitioners and dairy farm managers in the Ningxia region: Perform antimicrobial susceptibility testing before treatment, prioritize alternative agents (e.g., tigecycline, nitrofurantoin, and fosfomycin), and avoid florfenicol, which may co-select linezolid resistance; isolate affected cows, intensify disinfection of milking equipment, and regularly monitor environmental contamination; and establish a farm-level resistance database with routine screening for vancomycin and linezolid resistance genes (*vanF*, *optrA*, *cfr(A)*), and integrate the data into regional antimicrobial resistance surveillance networks for early warning and coordinated action. Additionally, provide on-farm training for veterinarians on rational drug use, horizontal gene transfer risks, and integrated mastitis control under the One Health framework.

## 5. Conclusions

Key findings showed that the bovine-derived *E. faecium* population in Ningxia (2019–2024) exhibited high genetic diversity and could be divided into two genetic subgroups and three clades. Within each subgroup, gene homology was high, while significant differences were observed between subgroups. Evolution of ARG and VF communities was dominated by temporal dynamics. This study underscores the importance of strengthening AMR monitoring and risk mitigation for mastitis-derived *E. faecium* in Ningxia, contributing to both the safety of the dairy production chain and the protection of public health.

## Figures and Tables

**Table 1:** Detection rate of *E. faecium* in Ningxia Hui Autonomous Region.

Group		Sample Size	Positive Samples	Positive Rate (%)
Year	2019	157	16	10.2
	2020	323	15	4.6
	2021	204	15	7.4
	2022	86	14	16.3
	2023	358	15	4.2
	2024	213	16	7.5
Area	Yinnan	539	45	8.3
	Yinchuan	457	30	6.6
	Yinbei	345	16	4.6

**Table 2:** Allelic profiles of novel sequence types identified in this study.

*E. faecium*	atpA	ddl	gdh	purK	gyd	pstS	adk	MLST
*E. faecium* *-1*	15	3	12	7	1	63	1	3084
*E. faecium* *-6*	20	124	1	3	1	117	1	3085
*E. faecium* *-13*	9	8	8	22	10	49	6	3086
*E. faecium* *-14*	9	136	12	3	1	1	1	3087
*E. faecium* *-15*	2	3	1	7	1	5	1	3088
*E. faecium* *-19*	5	8	8	17	8	19	6	3089
*E. faecium* *-25*	5	8	8	17	8	19	6	3089
*E. faecium* *-28*	9	8	8	22	10	49	6	3086
*E. faecium* *-38*	2	3	1	7	1	5	1	3088
*E. faecium* *-46*	5	3	12	7	10	134	1	3093
*E. faecium* *-47*	5	4	9	17	8	35	5	3094
*E. faecium* *-80*	5	4	9	17	8	35	5	3094
*E. faecium* *-90*	2	3	1	7	1	5	1	3088
*E. faecium* *-98*	5	3	1	3	6	1	1	3097
*E. faecium* *-105*	20	3	1	14	1	1	1	3098
*E. faecium* *-106*	5	3	1	3	6	1	1	3097

No duplicate samples originated from the same cow.

**Table 3:** Summary of AMOVA and MRPP Statistical Results for ARG Community Differences.

Method	Group	Comparison Group	Statistic (Fs/A)	*p*-Value	Significance
AMOVA	Areas	Yinbei-Yinchuan-Yinnan	0.358	0.997	ns
	Years (Adjacent Pairs)	2019 vs. 2020	2.181	0.056	ns
		2020 vs. 2021	3.952	0.012	
		2021 vs. 2022	0.225	0.967	ns
		2022 vs. 2023	0.918	0.479	ns
		2023 vs. 2024	3.859	0.001	
MRPP	Areas	Yinbei-Yinchuan-Yinnan	−0.007	0.987	ns
	Years (Overall)	2019–2024	0.051	0.001	

Note: ns = not significant (*p* > 0.05). Key nodes with significant temporal mutations are marked in the “Key Conclusion” column. The complete statistical data are provided in Supplementary Tables S5 and S6.

## Data Availability

The original data presented in this study are openly available in Mendeley Data at https://doi.org/10.17632/6w9dbwjc45.

## Supplementary Materials

The following supporting information can be downloaded at: https://www.mdpi.com/article/10.3390/microorganisms14071424/s1. The phylogenetic and pan-genome analysis data are available in the Mendeley Data repository at https://doi.org/10.17632/6w9dbwjc45. All other relevant data are contained within the manuscript and its supporting information files. Table S1: The information of *E. faecium* isolates used in the present study. Table S2: Data on resistance of *E. faecium* from milk. Table S3: Summary of mobile genetic elements data in dairy-derived *E. faecium*. Table S4: Drug resistance mutation sites in isolated strains. Table S5: Results of AMOVA analysis. Table S6: Results of MRPP analysis. Figure S1: The accumulation boxplot of Core/Pan genes. Figure S2: Minimum spanning tree based on multilocus sequence typing. Table S7: Raw data of whole-genome analysis. Table S8: Metadata on *E. faecium* genomes. References [56,57,58] are cited in the supplementary materials.

## Abbreviations

The following abbreviations are used in this manuscript:

*E. faecium*	*Enterococcus faecium*
MDR	Multidrug-resistant
AMR	Antimicrobial resistance
ANI	Average nucleotide identity
SNP	Single-nucleotide polymorphism
MLST	Multilocus sequence typing
COG	Clusters of orthologous groups
ARG	Antimicrobial resistance genes
MGE	Mobile genetic elements
MRPP	Multi-response permutation procedure
AMOVA	Analysis of molecular variance
PCoA	Principal coordinates analysis
MIC	Minimum inhibitory concentration
ST	Sequence types
IME	Integrative mobile elements
ICE	Integrative conjugative elements
T4SS	Type IV secretion system
