# Supplementary material for: Molecular Epidemiology, Phenotypic and Genomic Characterization of Multidrug-Resistant Enterococcus Faecium Isolated from Bovine Mastitis in Ningxia, China (2019–2024)
Source: Microorganisms. 2026 Jun 29;14(7):1424. doi: 10.3390/microorganisms14071424 (PMC13413553; doi:10.3390/microorganisms14071424)
Supplement: Supplementary file 1 [file microorganisms-14-01424-s001.zip › microorganisms-4338191-supplementary.pdf]

Supplementary Data Table S1. The information of *E. faecium* isolates used in the present study

Strain name	Isolation time (Year)	Isolation Province (Area)	MLST	Hemolysis	Genes
E.faecium-1	2019	Yinnan	3084*	γ	*poxtA*、*optrA*、*cfrA*
E.faecium-2	2019	Yinchuan	770	α	*poxtA, optrA, cfr(A)*
E.faecium-3	2019	Yinchuan	771	γ	*poxtA, optrA, cfr(A)*
E.faecium-4	2019	Yinnan	Unknown	α	*poxtA, optrA*
E.faecium-5	2019	Yinnan	Unknown	γ	*poxtA, optrA, cfr(A)*
E.faecium-6	2019	Yinnan	3085*	γ	*poxtA, optrA, cfr(A), fexB*
E.faecium-7	2019	Yinnan	Others	γ	*optrA, cfr(A)*
E.faecium-8	2019	Yinchuan	770	α	*optrA, cfr(E)*
E.faecium-10	2019	Yinchuan	Unknown	γ	*poxtA, optrA*
E.faecium-12	2019	Yinbei	Others	β	*poxtA, optrA, cfr(A)*
E.faecium-13	2019	Yinchuan	3086*	γ	*optrA, cfr(A)*
E.faecium-14	2019	Yinbei	3087*	β	*optrA, cfr(A)*
E.faecium-15	2019	Yinnan	3088*	β	*optrA, cfr(A)*
E.faecium-17	2019	Yinnan	Others	β	*poxtA, optrA, cfr(A)*
E.faecium-19	2019	Yinchuan	3089*	β	*poxtA, optrA, cfr(A)*
E.faecium-20	2019	Yinbei	771	β	*optrA, cfr(E)*
E.faecium-21	2020	Yinnan	Unknown	β	*optrA, cfr(A)*
E.faecium-22	2020	Yinnan	Unknown	β	*poxtA, optrA, cfr(A)*
E.faecium-24	2020	Yinchuan	27	β	*optrA, cfr(A)*
E.faecium-25	2020	Yinchuan	3089*	β	*poxtA, optrA, cfr(A)*
E.faecium-28	2020	Yinchuan	3086*	β	-
E.faecium-29	2020	Yinnan	Unknown	β	*poxtA, optrA, cfr(A)*
E.faecium-30	2020	Yinbei	Others	α	*poxtA, optrA, cfr(A)*
E.faecium-32	2020	Yinnan	Others	β	*poxtA, optrA, cfr(A)*
E.faecium-34	2020	Yinnan	80	γ	*optrA, cfr(A)*
E.faecium-35	2020	Yinbei	Unknown	α	*poxtA, optrA, cfr(A)*
E.faecium-37	2020	Yinnan	Unknown	β	*optrA, cfr(A)*
E.faecium-38	2020	Yinchuan	3088*	β	*optrA, cfr(A)*
E.faecium-40	2020	Yinchuan	770	α	*optrA, cfr(A)*
E.faecium-44	2020	Yinbei	Unknown	β	*optrA, cfr(A)*
E.faecium-45	2020	Yinnan	Unknown	γ	*poxtA, optrA, cfr(A)*
E.faecium-46	2021	Yinbei	3093*	β	*poxtA, optrA, cfr(A)*
E.faecium-47	2021	Yinnan	3094*	α	*optrA, cfr(A)*
E.faecium-48	2021	Yinbei	Others	β	*optrA, cfr(A)*
E.faecium-49	2021	Yinnan	18	β	*optrA, cfr(A)*
E.faecium-50	2021	Yinnan	17	β	*fexA, optrA, cfr(C)*
E.faecium-53	2021	Yinnan	296	γ	*poxtA, optrA, cfr(A)*
E.faecium-54	2021	Yinnan	27	β	*optrA, cfr(A)*
E.faecium-55	2021	Yinchuan	18	β	*optrA, cfr(A)*
E.faecium-56	2021	Yinchuan	18	β	*optrA, cfr(A)*
E.faecium-57	2021	Yinbei	Others	β	*poxtA, optrA, cfr(A)*
E.faecium-58	2021	Yinnan	Others	β	*optrA, cfr(A)*
E.faecium-59	2021	Yinchuan	18	β	*optrA, cfr(A)*
E.faecium-60	2021	Yinnan	771	β	*poxtA, optrA, cfr(A)*
E.faecium-61	2021	Yinnan	770	β	*optrA, cfr(A)*
E.faecium-62	2021	Yinnan	27	α	*optrA, cfr(A)*
E.faecium-64	2022	Yinnan	224	β	*poxtA, optrA, cfr(A)*
E.faecium-66	2022	Yinchuan	18	β	*optrA, cfr(A)*

Strain name	Isolation time (Year)	Isolation Province (Area)	MLST	Hemolysis	Genes
E.faecium-67	2022	Yinchuan	18	β	*optrA, cfr(A)*
E.faecium-68	2022	Yinnan	Others	β	*optrA, cfr(A)*
E.faecium-69	2022	Yinbei	Others	α	*optrA, cfr(A)*
E.faecium-70	2022	Yinnan	Others	β	*optrA, cfr(A)*
E.faecium-71	2022	Yinbei	18	β	*fexA, optrA, cfr(A)*
E.faecium-72	2022	Yinnan	80	β	*optrA, cfr(A)*
E.faecium-75	2022	Yinnan	Others	β	*optrA, cfr(A), fexB*
E.faecium-76	2022	Yinchuan	296	β	*poxtA, optrA, cfr(A)*
E.faecium-77	2022	Yinchuan	1532	β	*optrA, cfr(A), fexB*
E.faecium-80	2022	Yinnan	3094*	α	*optrA, cfr(A)*
E.faecium-81	2022	Yinnan	27	β	*optrA, cfr(A)*
E.faecium-83	2022	Yinchuan	Others	β	*optrA, cfr(A)*
E.faecium-85	2023	Yinbei	Others	β	*poxtA, optrA, cfr(A)*
E.faecium-88	2023	Yinchuan	771	β	*poxtA, optrA, cfr(A)*
E.faecium-90	2023	Yinchuan	3088*	β	*poxtA,optrA, cfr(A)*
E.faecium-94	2023	Yinnan	Others	β	*optrA, cfr(A)*
E.faecium-96	2023	Yinnan	1532	β	*optrA, cfr(A), fexB*
E.faecium-97	2023	Yinnan	80	β	*optrA, cfr(A)*
E.faecium-98	2023	Yinbei	3097*	β	*optrA, cfr(A), fexB*
E.faecium-100	2023	Yinchuan	Others	β	*optrA, cfr(A)*
E.faecium-101	2023	Yinnan	27	β	*optrA, cfr(A)*
E.faecium-102	2023	Yinchuan	80	β	*optrA, cfr(A)*
E.faecium-103	2023	Yinnan	Others	α	*poxtA, optrA, cfr(A)*
E.faecium-105	2023	Yinchuan	3098*	β	*poxtA, optrA, cfr(A)*
E.faecium-106	2023	Yinnan	3097*	α	*optrA, cfr(A), fexB*
E.faecium-107	2023	Yinnan	956	β	*fexA, optrA, cfr(A)*
E.faecium-108	2023	Yinchuan	956	α	*optrA, cfr(A)*
E.faecium-109	2024	Yinnan	18	γ	*optrA, cfr(A), fexB*
E.faecium-110	2024	Yinbei	Others	β	*poxtA,optrA, cfr(A)*
E.faecium-111	2024	Yinbei	17	γ	*fexA, optrA, cfr(A)*
E.faecium-112	2024	Yinnan	224	β	*poxtA, optrA, cfr(A)*
E.faecium-113	2024	Yinbei	27	α	*optrA, cfr(A)*
E.faecium-114	2024	Yinchuan	771	γ	*poxtA, optrA, cfr(A)*
E.faecium-115	2024	Yinnan	Unknown	α	*poxtA*
E.faecium-116	2024	Yinnan	Others	β	*optrA, cfr(A), fexB*
E.faecium-118	2024	Yinchuan	361	β	*poxtA, optrA, cfr(A)*
E.faecium-126	2024	Yinchuan	361	α	*poxtA, optrA, cfr(A)*
E.faecium-135	2024	Yinnan	361	α	*poxtA, optrA, cfr(A)*
E.faecium-137	2024	Yinchuan	296	α	*poxtA, optrA, cfr(A)*
E.faecium-146	2024	Yinchuan	27	γ	*optrA, cfr(A)*
E.faecium-147	2024	Yinnan	361	α	*poxtA, optrA, cfr(A)*
E.faecium-148	2024	Yinnan	361	α	*poxtA, optrA, cfr(A)*
E.faecium-150	2024	Yinnan	361	α	*poxtA, optrA, cfr(A)*

Note: "*" indicates a novel MLST sequence type. A total of 91 strains of *Enterococcus faecium* were isolated, and the strain numbers in the table do not correspond to the number of strains.

Supplementary Data Table S2. Data on resistance of *E. faecium* from milk

Strain name	LIN	TET	CIP	VAN	FOS	CFT	AMP	CHL	GEN	LNZ	SDZ	FFC	DOX	ERY
E.faecium-1	4	8	2	128	1024	1024	＞1024	64	＞1024	4	＞1024	16	2	4
E.faecium-2	4	4	2	64	512	256	512	64	＞1024	4	＞1024	16	2	2
E.faecium-3	2	4	2	1	4	<1	<4	4	4	2	512	8	2	4
E.faecium-4	4	1	0.5	1	8	<1	<4	4	32	4	256	16	2	4
E.faecium-5	4	4	4	4	1024	256	<4	32	＞1024	4	＞1024	16	4	4
E.faecium-6	4	4	0.5	0.25	16	16	<4	2	64	4	512	16	2	4
E.faecium-7	4	2	4	1	<1	<1	16	8	8	4	512	16	2	2
E.faecium-8	2	2	0.5	4	4	8	8	8	64	2	＞1024	8	1	8
E.faecium-10	2	1	2	<0.125	16	64	<4	4	64	2	512	8	32	2
E.faecium-12	2	1	4	128	256	512	＞1024	128	＞1024	2	＞1024	8	2	2
E.faecium-13	2	4	4	<0.125	4	16	<4	4	16	2	256	8	4	2
E.faecium-14	2	1	4	128	1024	128	＞1024	128	＞1024	2	＞1024	8	4	4
E.faecium-15	4	2	0.5	0.5	<1	<1	<4	8	64	4	128	16	8	16
E.faecium-17	2	2	2	128	1024	128	＞1024	128	＞1024	2	＞1024	8	8	2
E.faecium-19	2	2	2	0.5	32	32	<4	8	16	2	512	8	4	2
E.faecium-20	2	1	2	0.25	256	1024	32	8	64	2	＞1024	8	4	4
E.faecium-21	2	1	2	128	512	128	＞1024	128	＞1024	2	＞1024	8	2	2
E.faecium-22	4	1	0.5	128	128	256	＞1024	128	＞1024	4	＞1024	16	2	0.5
E.faecium-24	4	1	4	128	256	512	＞1024	128	＞1024	4	＞1024	16	2	4
E.faecium-25	4	1	32	64	512	512	1024	128	＞1024	4	＞1024	16	2	8
E.faecium-28	4	2	2	2	4	<1	<4	4	64	4	512	16	2	4
E.faecium-29	2	2	2	128	512	128	＞1024	128	＞1024	2	＞1024	8	0.5	2
E.faecium-30	2	2	4	<0.125	4	128	<4	2	16	2	512	8	4	2
E.faecium-32	2	1	2	64	128	512	＞1024	64	＞1024	2	＞1024	8	32	2
E.faecium-34	2	2	2	0.5	<1	<1	<4	2	8	2	512	8	2	2
E.faecium-35	2	16	0.5	1	16	<1	8	8	64	2	512	8	2	2

E.faecium-37	0.5	2	2	0.5	32	<1	<4	16	<4	0	16	0.5	2	4
E.faecium-38	4	2	2	2	256	128	<4	16	＞1024	4	＞1024	16	2	2
E.faecium-40	32	2	0.5	0.5	512	32	<4	16	16	32	512	128	4	2
E.faecium-44	2	2	4	128	256	256	256	64	＞1024	2	＞1024	8	2	2
E.faecium-45	2	2	32	0.5	8	<1	<4	8	<4	2	512	8	2	0.5
E.faecium-46	2	2	2	1	8	16	<4	16	<4	2	512	8	2	4
E.faecium-47	4	2	2	0.5	64	32	<4	32	16	4	512	16	0	4
E.faecium-48	4	1	2	8	128	128	16	16	＞1024	4	＞1024	16	4	2
E.faecium-49	4	4	0.5	2	>1024	256	<4	128	＞1024	4	＞1024	16	16	4
E.faecium-50	4	8	4	1	>1024	128	<4	32	512	4	1024	16	2	2
E.faecium-53	4	1	4	2	>1024	>1024	1024	8	＞1024	4	＞1024	16	8	2
E.faecium-54	4	1	4	1	4	32	<4	16	125	4	1024	16	4	0.5
E.faecium-55	2	1	4	0.125	256	256	16	16	125	2	＞1024	8	4	2
E.faecium-56	2	2	2	4	512	128	512	16	＞1024	2	＞1024	8	128	2
E.faecium-57	4	2	2	1	8	<1	<4	4	16	4	256	16	8	0.5
E.faecium-58	4	1	4	0.5	4	<1	<4	4	8	4	128	16	4	4
E.faecium-59	4	2	4	0.25	64	8	<4	2	＞1024	4	256	16	4	32
E.faecium-60	4	2	4	8	512	256	16	64	＞1024	4	＞1024	16	4	2
E.faecium-61	4	2	4	0.5	8	256	8	4	＞1024	4	＞1024	16	2	2
E.faecium-62	256	16	4	0.25	4	16	32	＜1	125	256	＞1024	1024	2	2
E.faecium-64	32	8	4	0.25	2	8	16	＜1	125	32	＞1024	128	16	4
E.faecium-66	8	16	2	4	>1024	>1024	<4	32	＞1024	8	＞1024	32	64	4
E.faecium-67	128	4	2	16	128	128	8	32	＞1024	128	＞1024	512	4	2
E.faecium-68	2	1	4	0.5	4	16	<4	8	64	2	＞1024	8	16	4
E.faecium-69	32	16	4	4	2	32	16	128	125	32	＞1024	128	32	4
E.faecium-70	4	32	2	16	128	256	＞1024	32	＞1024	4	＞1024	16	2	4
E.faecium-71	2	2	8	64	512	256	＞1024	32	＞1024	2	＞1024	8	4	4
E.faecium-72	2	2	1	0.25	16	16	16	＜1	125	2	＞1024	8	4	2

E.faecium-75	2	8	4	0.5	8	64	16	＜1	125	2	＞1024	8	8	2
E.faecium-76	2	8	4	0.25	8	32	16	1	125	2	＞1024	8	8	8
E.faecium-77	128	8	4	0.25	8	64	16	＜1	125	128	＞1024	512	16	2
E.faecium-80	16	4	2	64	512	512	＞1024	64	＞1024	16	＞1024	64	8	2
E.faecium-81	32	1	8	64	>1024	512	＞1024	64	＞1024	32	＞1024	128	8	4
E.faecium-83	32	16	4	64	4	128	＞1024	64	＞1024	32	128	128	16	2
E.faecium-85	2	16	8	32	>1024	256	＞1024	64	＞1024	2	＞1024	8	16	2
E.faecium-88	32	2	16	64	256	512	＞1024	64	＞1024	32	＞1024	128	4	2
E.faecium-90	2	2	32	64	>1024	512	＞1024	64	＞1024	2	＞1024	8	4	2
E.faecium-94	4	2	8	16	128	128	128	64	＞1024	4	＞1024	16	4	4
E.faecium-96	4	2	8	＜1	32	128	4	16	＞1024	4	＞1024	16	2	4
E.faecium-97	8	1	4	＜1	32	256	4	32	＞1024	8	＞1024	32	2	4
E.faecium-98	8	2	4	2	32	128	128	64	＞1024	8	＞1024	32	16	4
E.faecium-100	4	1	256	＜1	64	128	4	16	＞1024	4	＞1024	16	8	2
E.faecium-101	4	1	8	8	512	128	128	64	＞1024	4	＞1024	16	8	2
E.faecium-102	256	16	8	＜1	32	128	16	32	＞1024	256	＞1024	1024	16	2
E.faecium-103	8	4	4	64	128	256	512	64	＞1024	8	＞1024	32	16	2
E.faecium-105	8	8	16	＜1	128	128	4	16	＞1024	8	＞1024	32	8	2
E.faecium-106	32	8	4	64	>1024	512	＞1024	64	＞1024	32	＞1024	128	2	0.5
E.faecium-107	32	8	256	128	128	128	＞1024	64	＞1024	32	＞1024	128	256	4
E.faecium-108	32	16	32	64	64	32	＞1024	64	＞1024	32	256	128	32	32
E.faecium-109	2	8	8	64	64	512	＞1024	64	＞1024	2	＞1024	8	8	2
E.faecium-110	4	2	128	64	32	128	16	64	125	4	＞1024	16	128	2
E.faecium-111	4	2	2	64	32	256	16	64	125	4	＞1024	16	2	2
E.faecium-112	8	2	32	128	64	512	16	16	125	8	256	32	32	4
E.faecium-113	＞32	2	4	128	512	512	16	32	125	128	256	512	4	2
E.faecium-114	2	1	2	128	>1024	128	＞1024	64	＞1024	2	＞1024	8	2	0.5

E.faecium-115	8	1	2	64	128	128	＞1024	16	＞1024	8	512	32	2	4
E.faecium-116	8	1	2	2	32	256	＞1024	32	＞1024	8	512	32	2	32
E.faecium-118	＞32	2	2	128	32	128	＞1024	16	＞1024	256	1024	1024	2	8
E.faecium-126	32	8	128	128	32	128	＞1024	128	＞1024	32	512	128	128	256
E.faecium-135	8	16	16	64	64	128	＞1024	32	＞1024	8	＞1024	32	16	32
E.faecium-137	8	16	32	2	32	128	＞1024	32	＞1024	8	256	32	32	8
E.faecium-146	2	16	32	16	32	256	＞1024	16	＞1024	2	＞1024	8	32	8
E.faecium-147	4	16	2	＜1	256	128	＞1024	128	＞1024	4	128	16	2	2
E.faecium-148	2	8	32	128	128	512	＞1024	32	256	2	＞1024	8	32	4
E.faecium-150	8	2	16	8	32	128	＞1024	128	256	8	256	32	2	2

Note: To ensure that MICs could be determined for all isolates (including resistant strains), the tested concentration ranges were deliberately extended beyond the standard CLSI ranges. The two-fold serial dilution ranges for each antibiotic were as follows: lincomycin 0.5-256 µg/mL, tetracycline 0.5-256 µg/mL, ciprofloxacin 0.125-256 µg/mL, vancomycin 0.125-128 µg/mL, fosfomycin 1-1024 µg/mL, ceftiofur 1-1024 µg/mL, ampicillin 4-1024 µg/mL, chloramphenicol 1-128 µg/mL, gentamicin 4-1024 µg/mL, linezolid 2-256 µg/mL, trimethoprim 0.5-1024 µg/mL, florfenicol 0.5-128 µg/mL, doxycycline 0.5-128 µg/mL, and erythromycin 0.5-256 µg/mL. All concentration ranges covered the respective CLSI breakpoints, and the extended ranges allowed accurate determination of MICs even for highly resistant isolates. Each test was performed in triplicate using independent assays on three different days.

The MIC units for Lincomycin and Tetracycline are expressed in mg/ml, while the units for all other antibiotics are expressed in µg/ml.

Supplementary Data Table S3. Summary of mobile genetic elements data in dairy-derived*E. faecium*

Strain name	Plasmids													ISs											IME	ICE	Genes
	repUS15	rep1	rep2	repUS43	rep29	rep14b	rep17	repUS12	rep18b	repUS1	rep14a	rep18a	rep7a	IS66	IS3	IS256	IS30	ISL3	IS1182	IS200/IS605	IS1595	IS1380	IS6	IS982			
E.faecium-1	1	0	0	0	0	1	0	0	0	0	0	0	0	1	0	5	3	2	1	0	0	1	10	2	1	1	-
E.faecium-2	1	1	0	0	0	0	0	0	0	0	0	0	0	0	2	6	1	0	0	1	0	0	0	2	0	0	-
E.faecium-3	1	1	0	0	0	0	0	0	0	0	0	0	0	0	2	4	3	2	0	0	0	0	19	2	0	0	-
E.faecium-4	0	0	0	0	0	0	0	0	0	0	0	0	0	0	1	0	1	0	0	0	0	0	0	0	0	0	-
E.faecium-5	1	1	0	0	0	0	0	0	0	0	0	0	0	0	2	3	3	2	0	0	0	0	13	2	0	0	-
E.faecium-6	1	0	0	0	0	0	0	0	0	0	0	0	0	0	1	3	3	2	0	0	0	0	6	2	0	0	-
E.faecium-7	0	0	0	0	0	0	0	0	0	0	0	0	0	0	2	0	1	0	1	0	0	0	6	2	0	0	-
E.faecium-8	0	0	0	0	0	0	0	0	0	0	0	0	0	0	0	0	0	0	0	1	0	0	0	0	0	0	-
E.faecium-10	0	0	0	0	0	0	0	0	0	0	0	0	0	0	1	0	1	0	0	0	0	0	0	0	0	0	-
E.faecium-12	0	0	1	0	1	0	0	0	0	0	0	0	0	0	2	0	2	0	0	0	0	0	6	2	2	0	-
E.faecium-13	1	0	0	0	0	0	0	0	0	0	0	0	0	0	2	0	0	0	0	0	0	0	0	2	1	0	-
E.faecium-14	1	0	0	0	0	0	0	0	0	0	0	0	0	0	2	5	3	2	0	0	0	0	10	2	0	0	-
E.faecium-15	1	0	0	0	0	1	0	0	0	0	0	0	0	0	2	4	3	2	1	0	0	0	14	2	1	0	-
E.faecium-17	0	0	0	0	0	0	0	0	0	0	0	0	0	0	0	0	0	0	0	0	0	0	0	0	0	1	-
E.faecium-19	1	0	0	0	0	0	0	0	0	0	0	0	0	0	2	2	1	0	0	0	0	1	13	0	1	0	-
E.faecium-20	0	0	0	0	0	0	0	0	0	0	0	0	0	0	0	4	0	0	0	0	0	0	0	0	1	0	-
E.faecium-21	0	0	0	0	0	0	0	0	0	0	0	0	0	0	1	0	0	0	0	0	0	0	0	0	0	0	-
E.faecium-22	1	1	0	0	0	0	0	0	0	0	0	0	0	0	3	2	3	2	1	0	1	1	17	2	0	0	-
E.faecium-24	1	0	1	0	0	0	1	0	0	0	0	0	0	0	3	2	3	0	0	0	0	0	7	2	2	0	-
E.faecium-25	1	0	0	0	0	0	0	0	0	0	0	0	0	0	2	4	3	0	0	0	0	0	13	2	0	0	-
E.faecium-28	1	0	0	0	0	0	0	0	0	0	0	0	0	0	2	5	3	0	0	0	0	0	17	0	1	0	*tet(M)*
E.faecium-29	1	1	0	0	0	0	0	0	0	0	0	0	0	0	2	2	1	5	1	0	1	0	11	2	0	0	-
E.faecium-30	1	1	0	0	0	0	0	0	0	0	0	0	0	0	4	9	1	0	0	0	0	0	6	2	1	0	-
E.faecium-32	0	0	0	0	0	0	0	0	0	0	0	0	0	0	2	2	1	0	0	0	0	0	13	0	1	0	-

E.faecium-34	1	0	0	1	1	0	0	1	0	0	0	0	0	1	4	0	1	0	1	1	1	0	0	2	3	1	*tet(M)*
E.faecium-35	1	1	0	0	0	0	0	0	0	0	0	0	0	0	2	2	3	2	0	0	0	0	17	2	0	0	-
E.faecium-37	0	0	0	0	0	0	0	0	0	0	0	0	0	0	0	0	0	0	0	0	0	0	0	0	1	0	-
E.faecium-38	1	0	1	0	0	1	0	0	0	0	0	0	0	0	2	5	3	2	1	0	0	0	11	2	1	0	-
E.faecium-40	1	0	0	0	0	0	0	0	0	0	0	0	0	0	0	0	0	0	0	1	0	0	0	0	0	0	-
E.faecium-44														0	1	0	0	0	0	0	0	0	0	0	0	0	-
E.faecium-45	1	1	0	0	0	0	0	0	0	0	0	0	0	0	2	2	3	2	0	0	0	0	19	2	1	0	-
E.faecium-46	1	0	0	0	0	1	0	0	0	0	0	0	0	0	2	7	3	2	0	0	0	0	5	0	1	0	-
E.faecium-47	1	0	1	1	0	0	0	0	0	0	0	1	0	0	2	5	1	2	1	0	1	0	11	2	0	1	*tet(M)*
E.faecium-48	1	0	1	1	0	1	1	0	0	0	0	0	0	1	5	8	1	4	1	1	1	0	5	2	4	1	-
E.faecium-49	1	0	0	1	1	1	0	1	1	0	0	0	0	1	5	8	3	3	2	1	1	0	12	2	5	2	*aac(6')-Ii*、*tet(M)*
E.faecium-50	1	0	0	1	1	1	0	1	1	0	0	0	0	1	5	9	4	2	1	1	1	0	12	2	2	2	-
E.faecium-53	0	0	0	0	1	0	0	0	0	0	0	0	0	0	2	6	1	0	0	0	0	0	0	2	0	0	-
E.faecium-54	1	0	1	1	0	0	1	0	0	0	0	0	0	0	3	4	3	0	1	0	1	0	15	2	2	1	*tet(L)*、*tet(M)*
E.faecium-55	0	0	0	1	1	1	0	1	1	0	0	0	0	1	5	9	3	3	2	1	1	0	12	2	3	2	*aac(6')-Ii*
E.faecium-56	1	0	0	1	1	1	0	1	1	0	0	0	0	1	5	9	3	3	2	1	1	0	12	2	3	2	*aac(6')-Ii*
E.faecium-57	1	0	0	0	0	0	0	0	0	0	0	0	0	0	3	4	1	0	0	0	0	0	17	2	0	0	-
E.faecium-58	1	1	1	0	0	0	0	0	0	0	0	0	1	0	2	6	2	0	2	0	1	0	5	0	1	0	-
E.faecium-59	1	0	0	1	1	1	0	1	1	0	0	0	0	1	5	8	3	3	2	1	1	0	12	2	4	2	*aac(6')-Ii*、*tet(L)*
E.faecium-60	0	1	1	1	0	1	1	0	0	0	0	0	0	1	4	7	1	2	1	0	1	0	6	0	1	2	-
E.faecium-61	1	1	0	0	0	0	0	0	0	0	0	0	0	0	3	4	1	1	0	0	0	0	10	2	1	0	-
E.faecium-62	1	0	1	1	0	0	1	0	0	0	0	0	0	0	3	4	3	0	1	0	0	0	8	2	1	1	*tet(M)*
E.faecium-64	1	0	0	0	0	0	0	0	0	1	0	0	0	0	3	4	3	0	1	0	0	0	8	2	0	2	-
E.faecium-66	1	0	0	1	1	1	0	1	1	0	0	0	0	0	2	7	2	2	1	0	0	0	14	2	3	2	*aac(6')-Ii*

E.faecium-67	1	0	0	1	1	1	0	1	1	0	0	0	0	1	5	9	3	3	2	1	1	0	12	2	4	2	*aac(6')-Ii*
E.faecium-68	1	0	1	0	0	0	0	0	0	0	0	0	0	0	2	3	4	2	0	0	0	0	6	2	1	0	-
E.faecium-69	1	1	1	1	0	0	0	0	0	0	0	0	0	0	2	4	4	2	0	0	1	0	6	2	0	1	*aph(3')-lll*、*emm(B)*、*tet(M)*、*tet(L)*
E.faecium-70	1	0	0	1	1	1	0	1	1	0	1	0	0	1	5	10	3	5	2	1	1	0	8	2	5	1	*aac(6')-Ii*
E.faecium-71	1	0	0	1	1	0	0	1	1	0	0	0	0	1	5	9	3	3	2	1	1	0	12	2	4	3	*aac(6')-Ii*、*tet(L)*
E.faecium-72	1	0	0	1	1	0	0	1	0	0	0	0	0	1	5	10	1	5	1	1	1	0	11	2	3	1	*tet(L)*
E.faecium-75	1	1	1	0	1	1	0	0	0	0	0	0	0	0	4	5	2	3	1	1	1	0	19	2	1	0	*tet(M)*
E.faecium-76	0	0	0	0	1	0	0	0	0	0	0	0	0	0	2	6	1	0	0	0	0	0	0	2	0	0	-
E.faecium-77	1	1	1	0	1	0	0	0	1	0	0	0	0	0	2	2	2	0	1	0	1	0	10	0	0	0	-
E.faecium-80	1		1	1	0	0	0	0	0	0	0	0	0	0	2	5	1	2	1	0	1	0	11	2	0	1	*tet(L)*
E.faecium-81	1	0	1	0	0	0	1	0	0	0	0	0	0	0	3	2	3	0	1	0	1	1	7	0	2	0	*tet(M)*
E.faecium-83														0	1	0	0	0	0	0	0	0	0	0	0	0	-
E.faecium-85	1	1	0	0	0	0	0	0	0	0	0	0	0	0	2	6	3	0	0	0	0	0	17	2	1	0	-
E.faecium-88	0	1	1	1	0	1	1	0	0	0	0	0	0	1	4	7	1	2	1	0	1	0	6	0	1	2	-
E.faecium-90	1	0	1	0	0	1	0	0	0	0	0	0	0	0	2	5	3	2	1	0	0	0	11	2	0	0	-
E.faecium-94														0	0	0	0	0	0	0	0	0	0	0	0	1	-
E.faecium-96	1	1	1	0	1	0	0	0	1	0	0	0	0	0	2	2	2	0	1	0	1	0	0	2	0	0	-
E.faecium-97	1	0	0	1	1	0	0	1	0	0	1	0	0	1	5	10	1	5	1	1	1	0	6	2	2	1	*tet(M)*
E.faecium-98	1	0	1	1	1	0	1	0	0	0	0	0	0	0	3	7	4	0	1	1	1	1	9	2	0	0	-
E.faecium-100														0	2	4	1	0	1	0	0	0	6	2	1	0	-
E.faecium-101	1	0	1	1	0	0	1	0	0	0	0	0	0	0	3	4	3	0	1	0	1	0	13	2	1	1	*tet(L)*、*tet(M)*
E.faecium-102	1	0	0	1	1	0	0	1	0	0	1	0	0	1	5	9	1	5	1	1	1	0	6	2	2	1	*tet(M)*

E.faecium-103	1	1	0	0	0	0	0	0	0	0	0	0	0	0	2	4	1	2	0	0	0	0	10	2	0	0	-
E.faecium-105	1	0	0	1	0	0	0	0	0	1	0	0	0	0	3	7	3	2	1	0	1	1	11	2	0	0	-
E.faecium-106	1	0	0	1	1	0	1	0	0	0	0	0	0	0	3	8	4	0	1	1	1	1	7	2	1	0	-
E.faecium-107	1	0	1	1	0	0	0	0	0	0	0	0	0	0	3	7	2	1	1	1	1	1	20	2	1	1	*tet(L)*
E.faecium-108	1	0	1	1	0	0	0	0	0	0	0	0	0	0	3	7	2	1	1	1	1	1	20	2	1	1	*tet(M)*
E.faecium-109	1	0	0	1	1	0	1	1	1	0	0	0	0	1	5	8	4	3	2	1	1	0	12	2	3	1	*aac(6')-Ii*
E.faecium-110	0	1	1	0	0	0	0	0	0	1	0	0	0	0	4	7	2	1	1	0	1	0	18	2	0	1	-
E.faecium-111	1	0	0	1	1	0	1	1	0	0	0	0	0	1	5	9	3	2	1	1	1	0	12	2	1	2	-
E.faecium-112	1	1	1	0	1	1	0	0	0	0	0	0	0	0	2	7	4	0	0	0	0	0	13	2	1	1	*tet(L)*、*tet(M)*
E.faecium-113	1	1	1	1	0	0	1	0	0	1	0	0	0	0	3	6	3	0	1	0	0	0	14	2	1	2	*erm(B)*、*tet(M)*
E.faecium-114	0	1	1	0	0	0	1	0	0	0	0	0	0	0	4	6	1	2	1	0	1	0	6	2	2	0	*tet(L)*
E.faecium-115	0	0	0	0	0	0	0	0	0	0	0	0	0	0	1	0	0	0	0	0	0	0	13	0	1	0	-
E.faecium-116	1	1	1	1	0	0	0	0	0	0	0	0	0	0	2	2	0	0	0	0	0	0	10	2	0	1	*tet(M)*
E.faecium-118	1	0	0	0	0	0	0	0	0	0	0	0	0	0	3	6	1	0	0	0	0	0	13	2	0	0	-
E.faecium-126	1	0	0	0	0	0	0	0	0	0	0	0	0	0	3	4	1	0	0	0	0	0	11	2	0	0	-
E.faecium-135	1	0	0	0	0	0	0	0	0	0	0	0	0	0	3	4	1	0	0	0	0	0	14	2	0	0	-
E.faecium-137	0	0	0	0	0	1	0	0	0	0	0	0	0	0	2	6	1	0	0	0	0	0	0	2	0	0	-
E.faecium-146	1	0	1	1	1	0	1	0	0	0	0	0	0	0	3	2	3	0	0	0	1	0	13	2	0	1	*tet(L)*、*tet(M)*
E.faecium-147	1	0	0	0	0	0	0	0	0	0	0	0	0	0	3	4	1	0	0	0	0	0	14	2	0	0	-
E.faecium-148	1	0	0	0	0	0	0	0	0	0	0	0	0	0	2	4	1	0	0	0	0	0	14	2	0	0	-
E.faecium-150	1	0	0	0	0	0	0	0	0	0	0	0	0	0	3	4	1	0	0	0	0	0	14	2	0	0	-

Supplementary Data Table S4. Drug resistance mutation sites in isolated strains

Strain name	MLST	Ponint mutations leading to AMR		
		gyrA	parC	pbp5
E.faecium-1	3084*	0	0	0
E.faecium-2	770	0	0	1
E.faecium-3	771	0	0	1
E.faecium-4	Unknown	0	0	0
E.faecium-5	Unknown	0	0	1
E.faecium-6	3085*	0	0	1
E.faecium-7	Others	0	0	1
E.faecium-8	770	0	0	0
E.faecium-10	Unknown	0	0	0
E.faecium-12	Others	0	0	0
E.faecium-13	3086*	0	0	1
E.faecium-14	3087*	0	0	1
E.faecium-15	3088*	0	0	1
E.faecium-17	Others	0	0	1
E.faecium-19	3089*	0	0	0
E.faecium-20	771	0	0	0
E.faecium-21	Unknown	0	0	1
E.faecium-22	Unknown	0	0	1
E.faecium-24	27	0	0	1
E.faecium-25	3089*	0	0	0
E.faecium-28	3086*	0	0	1
E.faecium-29	Unknown	0	0	1
E.faecium-30	Others	0	0	1
E.faecium-32	Others	0	0	0
E.faecium-34	80	1	1	1
E.faecium-35	Unknown	0	0	1
E.faecium-37	Unknown	0	0	1
E.faecium-38	3088*	0	0	1
E.faecium-40	770	0	0	1
E.faecium-44	Unknown	0	0	1
E.faecium-45	Unknown	0	0	1
E.faecium-46	3093*	0	0	1
E.faecium-47	3094*	0	0	1
E.faecium-48	Others	1	1	1
E.faecium-49	18	1	1	1
E.faecium-50	17	1	1	1
E.faecium-53	296	0	0	0
E.faecium-54	27	0	0	1
E.faecium-55	18	1	1	1
E.faecium-56	18	1	1	1
E.faecium-57	Others	0	0	0
E.faecium-58	Others	0	0	1
E.faecium-59	18	1	1	1
E.faecium-60	771	0	0	1
E.faecium-61	770	0	0	1
E.faecium-62	27	0	0	1
E.faecium-64	224	0	0	1
E.faecium-66	18	1	1	1

Strain name	MLST	Ponint mutations leading to AMR		
		gyrA	parC	pbp5
E.faecium-67	18	1	1	1
E.faecium-68	Others	0	0	1
E.faecium-69	Others	0	0	1
E.faecium-70	Others	1	1	1
E.faecium-71	18	1	1	1
E.faecium-72	80	1	1	1
E.faecium-75	Others	0	0	1
E.faecium-76	296	0	0	0
E.faecium-77	1532	0	0	1
E.faecium-80	3094*	0	0	0
E.faecium-81	27	0	0	1
E.faecium-83	Others	0	0	1
E.faecium-85	Others	0	0	0
E.faecium-88	771	0	0	1
E.faecium-90	3088*	0	0	1
E.faecium-94	Others	0	0	1
E.faecium-96	1532	0	0	1
E.faecium-97	80	1	1	1
E.faecium-98	3097*	1	1	1
E.faecium-100	Others	0	0	1
E.faecium-101	27	0	0	1
E.faecium-102	80	1	1	1
E.faecium-103	Others	0	0	1
E.faecium-105	3098*	0	0	1
E.faecium-106	3097*	1	1	1
E.faecium-107	956	0	0	1
E.faecium-108	956	0	0	1
E.faecium-109	18	1	1	1
E.faecium-110	Others	0	0	1
E.faecium-111	17	1	1	1
E.faecium-112	224	0	0	0
E.faecium-113	27	0	0	1
E.faecium-114	771	0	0	1
E.faecium-115	Unknown	0	0	0
E.faecium-116	Others	0	0	1
E.faecium-118	361	0	0	0
E.faecium-126	361	0	0	0
E.faecium-135	361	0	0	0
E.faecium-137	296	0	0	0
E.faecium-146	27	0	0	1
E.faecium-147	361	0	0	0
E.faecium-148	361	0	0	0
E.faecium-150	361	0	0	0

Note: "*" indicates a novel MLST sequence type.

Supplementary Data Table S5. Results of AMOVA analysis

		Vs group	SS	df	MS	Fs	p-value
CARD	Areas	Yinbei-Yinchuan-Yinnan	0.020 (2.48345)	2(88)	0.010 (0.0282211)	0.358	0.997
	Years	2019-2020-2021-2022-2023-2024	0.386 (2.11729)	5(85)	0.077 (0.0249093)	3.102	-
		2019-2020	0.071 (0.949667)	1(29)	0.071 (0.0327472)	2.181	0.056
		2019-2021	0.144 (0.967613)	1(29)	0.144 (0.033366)	4.304	0.001
		2019-2022	0.151 (0.979306)	1(28)	0.151 (0.0349752)	4.306	0.001
		2019-2023	0.179 (1.00037)	1(29)	0.179 (0.0344956)	5.176	-
		2019-2024	0.083 (1.21473)	1(30)	0.083 (0.0404909)	2.038	0.046
		2020-2021	0.059 (0.420083)	1(28)	0.059 (0.015003)	3.952	0.012
		2020-2022	0.059 (0.431776)	1(27)	0.059 (0.0159917)	3.701	0.011
		2020-2023	0.068 (0.452843)	1(28)	0.068 (0.016173)	4.192	0.003
		2020-2024	0.0494182 (0.667196)	1(29)	0.049 (0.0230067)	2.148	0.070
		2021-2022	0.004 (0.449722)	1(27)	0.004 (0.0166564)	0.225	0.967
		2021-2023	0.018 (0.470789)	1(28)	0.018 (0.0168139)	1.064	0.368
		2021-2024	0.074 (0.685142)	1(29)	0.074 (0.0236256)	3.122	0.012
		2022-2023	0.0163976 (0.482482)	1(27)	0.016 (0.0178697)	0.918	0.479
		2022-2024	0.079 (0.696834)	1(28)	0.079 (0.0248869)	3.172	0.018
		2023-2024	0.096 (0.717902)	1(29)	0.096 (0.0247552)	3.859	0.001
VF	Areas	Yinbei-Yinchuan-Yinnan	0.009 (0.862804)	2(88)	0.004 (0.00980459)	0.440	0.965
	Years	2019-2020-2021-2022-2023-2024	0.124 (0.747874)	5(85)	0.025 (0.00879852)	2.809	
		2019-2020	0.027 (0.354346)	1(29)	0.027 (0.0122188)	2.188	0.085
		2019-2021	0.045 (0.339732)	1(29)	0.045 (0.0117149)	3.872	0.002
		2019-2022	0.046 (0.346602)	1(28)	0.046 (0.0123787)	3.700	0.003
		2019-2023	0.058 (0.342199)	1(29)	0.058 (0.0118)	4.873	
		2019-2024	0.0268 (0.453682)	1(30)	0.026 (0.0151227)	1.742	0.095
		2020-2021	0.022 (0.149734)	1(28)	0.022 (0.00534766)	4.151	0.015
		2020-2022	0.022 (0.156604)	1(27)	0.022 (0.00580016)	3.712	0.021
		2020-2023	0.030 (0.152201)	1(28)	0.030 (0.00543575)	5.436	0.005
		2020-2024	0.017	1(29)	0.017	1.884	0.088

Vs group	SS	df	MS	Fs	p-value
	(0.263684)		(0.00909255)		
2021-2022	0.001 (0.141991)	1(27)	0.001 (0.00525892)	0.126	0.995
2021-2023	0.00281341 (0.137588)	1(28)	0.003 (0.00491384)	0.573	0.725
2021-2024	0.023 (0.249071)	1(29)	0.023 (0.00858864)	2.630	0.033
2022-2023	0.003 (0.144457)	1(27)	0.003 (0.00535027)	0.606	0.701
2022-2024	0.020 (0.25594)	1(28)	0.020 (0.00914073)	2.134	0.055
2023-2024	0.026 (0.251537)	1(29)	0.026 (0.0086737)	3.047	0.015

Supplementary Data Table S6. Results of MRPP analysis

		Group	A	Observed delta	Expected delta	Significance
CARD	Areas	Yinbei-Yinchuan-Yinnan	-0.007	8.444	8.387	0.987
	Years	2019-2020-2021-2022-2023-2024	0.051	7.96	8.387	0.001
VF	Areas	Yinbei-Yinchuan-Yinnan	-0.005	9.062	9.015	0.919
	Years	2019-2020-2021-2022-2023-2024	0.051	8.554	9.015	0.001

Supplementary Figure S1. The accumulation boxplot of Core/Pan genes

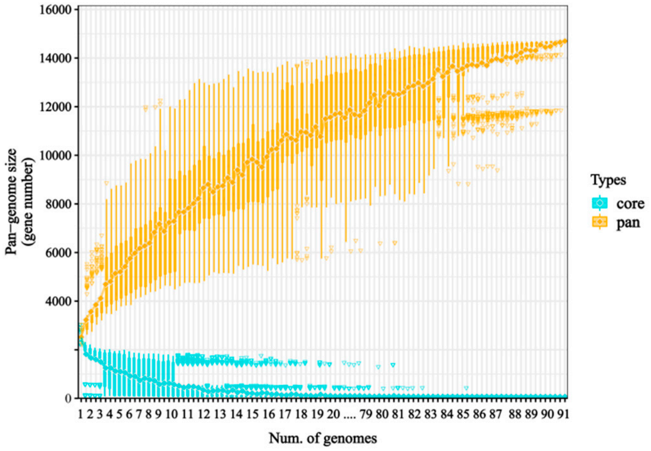

Supplementary Figure S2. Minimum spanning tree based on multilocus sequence typing

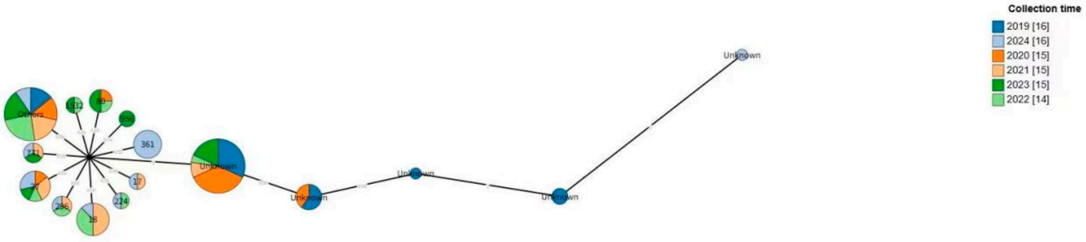

Supplementary Data Table S7. Raw data of whole-genome analysis

Strain name	Total clean reads	Total clean bases (bp)	Q30 (%)	GC (%)	Genome size (bp))	Contig count	N50 (bp)	Gene count	Avg. coverage (×)	L50
E.faecium-1	7,765,754	1,159,964,921	94.98	38.96	2,750,092	95	79,080	2,682	422	13
E.faecium-2	7,228,154	1,080,024,059	95.16	39.14	2,501,677	33	177,218	2,510	450	6
E.faecium-3	7,205,588	1,076,293,620	95.13	39.01	2,686,327	30	225,628	2,582	401	4
E.faecium-4	7,410,252	1,107,954,715	95.30	38.45	2,699,788	54	86,092	2,557	410	10
E.faecium-5	6,880,570	1,029,052,254	94.92	39.00	2,560,966	35	183,558	2,363	418	5
E.faecium-6	6,909,658	1,033,601,389	94.97	38.76	2,741,903	54	187,818	2,662	377	6
E.faecium-7	7,372,598	1,101,343,424	95.11	38.72	2,560,537	37	143,753	2,492	430	6
E.faecium-8	8,110,218	1,212,206,664	94.62	37.71	2,958,204	42	241,259	2,673	410	5
E.faecium-10	8,746,902	1,305,887,040	94.43	38.18	2,700,255	48	119,919	2,561	484	8
E.faecium-12	6,018,420	899,167,465	95.14	39.03	2,566,586	43	149,506	2,497	350	6
E.faecium-13	8,600,146	1,284,560,887	95.22	38.77	2,635,562	38	274,835	2,545	487	4
E.faecium-14	7,341,914	1,096,309,958	95.21	38.80	2,652,029	78	110,532	2,601	413	9
E.faecium-15	7,221,384	1,078,326,409	95.46	38.68	2,692,852	59	112,582	2,596	400	8
E.faecium-17	8,005,458	1,196,368,364	95.17	38.91	2,739,882	34	148,999	2,619	437	7
E.faecium-19	7,120,162	1,062,723,668	95.12	38.83	2,761,093	56	154,622	2,763	385	7
E.faecium-20	25,041,266	3,741,977,723	96.49	37.48	2,954,918	52	164,405	2,712	1266	6
E.faecium-21	7,151,314	1,065,572,579	95.33	38.71	2,589,965	22	198,460	2,508	411	4
E.faecium-22	7,893,266	1,176,801,290	95.33	39.03	2,762,628	36	177,135	2,654	426	5
E.faecium-24	6,814,326	1,016,301,223	95.16	38.61	2,839,384	108	63,461	2,816	358	13
E.faecium-25	7,774,374	1,160,849,253	95.15	38.93	2,764,343	57	119,485	2,764	420	8
E.faecium-28	6,954,522	1,038,994,651	95.05	38.84	2,802,426	43	274,835	2,715	371	4
E.faecium-29	7,060,604	1,052,583,170	95.63	38.89	2,518,463	29	274,494	2,435	418	4
E.faecium-30	9,326,422	1,389,882,669	94.99	38.92	2,673,056	73	98,673	2,618	520	9
E.faecium-32	6,949,180	1,037,653,464	95.12	38.53	2,737,828	49	100,264	2,648	379	8
E.faecium-34	7,197,172	1,074,269,520	95.42	38.48	2,828,107	101	91,893	2,780	380	18
E.faecium-35	7,498,540	1,118,693,756	95.13	38.98	2,454,332	29	258,601	2,356	456	4
E.faecium-37	7,519,246	1,120,430,862	95.71	38.84	2,549,744	23	208,963	2,449	440	5
E.faecium-38	7,431,410	1,107,722,520	95.20	38.77	2,702,600	84	95,964	2,598	410	10
E.faecium-40	7,456,002	1,112,839,863	95.16	38.56	2,600,142	29	243,660	2,482	428	3
E.faecium-44	7,850,590	1,172,072,717	95.09	38.95	2,587,569	29	198,492	2,502	453	5
E.faecium-45	7,044,612	1,053,609,499	94.97	39.19	2,679,100	34	178,150	2,572	393	6
E.faecium-46	7,256,134	1,085,239,118	94.86	38.73	2,695,758	83	86,203	2,616	403	10
E.faecium-47	8,572,948	1,280,597,939	94.85	38.90	2,793,087	109	71,344	2,769	459	14
E.faecium-48	8,063,646	1,203,450,390	95.31	38.71	2,724,600	138	49,818	2,662	442	19
E.faecium-49	8,370,822	1,249,520,660	95.12	38.68	2,736,249	72	63,555	2,657	457	18
E.faecium-50	8,332,680	1,243,945,394	95.24	38.51	2,856,103	84	62,296	2,789	436	20
E.faecium-53	7,531,598	1,125,429,596	95.16	39.05	2,639,340	60	91,420	2,583	426	11
E.faecium-54	7,569,772	1,130,527,220	94.81	39.27	2,733,021	105	51,393	2,656	413.64	15
E.faecium-55	8,215,654	1,226,699,743	94.78	39.11	2,718,053	167	42,588	2,633	451.31	19
E.faecium-56	6,913,788	1,032,558,783	95.11	38.85	2,730,197	153	43,491	2,648	378.20	18
E.faecium-57	7,316,704	1,093,223,626	94.77	39.43	2,674,970	85	92,602	2,609	408.69	11
E.faecium-58	7,439,936	1,111,355,901	94.87	39.17	2,746,900	98	123,557	2,691	404.62	9
E.faecium-59	7,929,196	1,184,672,257	95.15	38.93	2,722,071	151	43,739	2,635	435.27	18
E.faecium-60	8,640,436	1,290,183,488	94.99	38.98	2,739,074	105	88,341	2,740	471.05	12
E.faecium-61	7,360,920	1,100,200,281	94.90	39.04	2,627,009	76	95,016	2,546	418.90	10
E.faecium-62	7,283,100	1,088,046,173	94.93	38.85	2,745,863	102	69,114	2,681	396.32	12
E.faecium-64	7,524,824	1,124,812,448	95.15	38.67	2,994,744	108	66,990	3,000	375.58	13

E.faecium-66	6,383,180	954,137,978	94.90	38.52	2,735,254	148	43,857	2,654	348.85	18
E.faecium-67	8,157,650	1,216,315,451	95.24	38.68	2,732,821	152	43,857	2,650	445.08	18
E.faecium-68	7,739,980	1,156,333,843	95.27	38.74	2,748,239	77	138,500	2,680	420.80	8
E.faecium-69	7,311,788	1,090,848,701	95.09	38.81	2,777,779	77	96,032	2,708	392.70	9
E.faecium-70	7,948,270	1,186,053,902	95.32	38.55	2,779,433	87	73,373	3,029	373.07	17
E.faecium-71	6,702,818	1,000,686,217	95.33	38.91	2,762,563	151	43,271	2,688	362.22	18
E.faecium-72	7,236,098	1,079,339,188	95.18	38.65	2,835,518	148	45,015	2,795	380.68	22
E.faecium-75	7,904,200	1,180,245,013	95.08	39.02	2,652,360	124	58,932	2,581	445.03	15
E.faecium-76	7,393,986	1,103,866,347	95.22	39.14	2,637,673	61	91,420	2,576	418.57	11
E.faecium-77	8,531,198	1,272,218,692	95.26	38.90	2,732,270	82	100,704	2,676	465.56	10
E.faecium-80	7,155,162	1,067,716,889	95.09	38.98	2,795,202	119	49,558	2,776	382.01	14
E.faecium-81	10,166,206	1,515,507,702	95.39	39.11	2,811,387	108	59,452	2,795	539.01	15
E.faecium-83	7,188,058	1,073,585,735	95.23	39.28	2,561,947	43	131,156	2,453	419.05	7
E.faecium-85	7,639,378	1,141,775,348	95.02	39.42	2,615,306	61	127,850	2,557	436.58	8
E.faecium-88	7,336,984	1,097,269,483	94.88	38.87	2,734,994	103	88,341	2,738	401.28	12
E.faecium-90	7,031,792	1,049,984,258	95.13	39.26	2,693,093	95	75,626	2,585	389.89	11
E.faecium-94	8,204,750	1,227,639,511	94.32	39.14	2,618,306	30	157,527	2,481	468.80	7
E.faecium-96	7,171,572	1,072,441,588	94.99	39.26	2,727,988	98	86,855	2,665	393.09	11
E.faecium-97	7,593,794	1,135,659,718	94.75	38.94	2,833,443	146	41,277	2,793	400.75	21
E.faecium-98	10,518,692	1,571,745,047	95.21	39.59	2,701,677	169	49,240	2,636	581.68	19
E.faecium-100	7,865,304	1,177,683,595	94.93	39.70	2,458,574	77	77,839	2,382	478.96	11
E.faecium-101	7,661,086	1,142,662,169	95.33	38.95	2,810,012	111	56,156	2,775	406.64	15
E.faecium-102	7,436,762	1,111,331,152	95.26	39.16	2,828,879	146	51,345	2,791	392.88	19
E.faecium-103	7,091,718	1,058,789,799	95.19	39.81	2,750,859	91	67,213	2,697	384.93	13
E.faecium-105	8,327,250	1,243,421,736	95.21	38.52	2,809,693	77	159,420	2,746	442.60	5
E.faecium-106	8,028,270	1,198,403,788	95.27	38.41	2,753,301	116	101,038	2,712	435.27	11
E.faecium-107	7,884,726	1,178,032,872	95.11	38.48	2,669,540	113	63,253	2,629	441.28	11
E.faecium-108	6,572,358	985,007,817	94.98	38.54	2,669,591	111	63,253	2,629	369.08	10
E.faecium-109	7,474,310	1,097,109,888	95.99	39.09	2,755,782	141	61,432	2,676	389.21	22
E.faecium-110	6,654,796	1,095,565,841	95.11	39.80	2,698,520	133	58,690	2,706	379.04	24
E.faecium-111	6,284,184	1,040,153,252	95.06	40.05	2,778,375	136	59,334	2,714	398.4	29
E.faecium-112	7,805,678	1,167,076,974	95.09	40.10	2,778,869	103	50,165	2,981	391.8	19
E.faecium-113	7,107,526	1,063,948,332	94.94	40.10	2,821,556	105	51,473	2,780	377.1	16
E.faecium-114	6,942,230	1,038,986,902	95.38	40.32	2,679,233	90	72,236	2,671	387.8	13
E.faecium-115	6,473,170	968,818,652	95.05	39.08	2,875,569	74	92,733	3,130	315.0	9
E.faecium-116	30,360,456	4,542,171,027	96.15	40.29	2,719,771	87	90,795	2,623	1,670.1	10
E.faecium-118	6,849,138	1,025,384,461	94.97	40.53	2,634,767	98	68,550	2,589	389.2	15
E.faecium-126	6,390,064	957,298,372	94.99	40.81	2,635,012	85	78,858	2,594	363.3	12
E.faecium-135	50,182,562	7,515,157,814	95.85	41.48	2,589,073	93	80,775	2,538	2,902.7	11
E.faecium-137	28,827,350	4,315,264,721	96.01	41.72	2,634,110	88	79,317	2,652	1,638.0	13
E.faecium-146	9,441,108	1,409,351,277	94.95	38.34	2,721,110	87	75,016	2,666	517.9	12
E.faecium-147	8,813,840	1,311,772,695	95.29	38.28	2,650,169	63	106,531	2,613	495.0	9
E.faecium-148	9,147,088	1,366,670,128	94.79	38.62	2,650,533	65	106,281	2,612	515.7	9
E.faecium-150	9,031,084	1,346,229,460	95.43	38.70	2,649,793	63	106,281	2,611	508.0	9

Supplementary Data Table S8. Metadata on *E. faecium* genomes

Order on tree	Clade	Strain designation	Year of Isolation	Country	ST[b]	CC[c]	Source	Isolation site	Category	Source
1	B	EnGen0003	2001	IRL	163		Non-hospitalized individual	Feces	Non-hospitalized individual	[1]
2	B	Com12	2006	USA	107		Non-hospitalized individual	Feces	Non-hospitalized individual	[2]
3	B	EnGen0056	2000	NLD	327		Hospitalized patient	Blood	Clinical Isolate	[1]
4	B	EnGen0047	2004	NLD	328		Hospitalized patient	Blood	Clinical Isolate	[1]
5	B	1_141_733	2005	USA	327		Hospitalized patient	Wound	Clinical Isolate	[2]
6	B	EnGen0038	2006	NLD	331		Hospitalized patient	Blood	Clinical Isolate	[1]
7	B	Com15	2007	USA	583		Non-hospitalized individual	Feces	Non-hospitalized individual	[2]
8	B	EnGen0042	2001	ESP	289		Hospitalized patient	Feces	Hosp_Feces	[1]
9	B	E980	1998	NLD	94		Non-hospitalized individual	Feces	Non-hospitalized individual	[3]
10	B	EnGen0033	2000	DEU	299		Hospitalized patient	Blood	Clinical Isolate	[1]
11	B	EnGen0015	1998	NLD	61		Non-hospitalized individual	Feces	Non-hospitalized individual	[1]
12	B	EnGen0028	1956	NOR	75		Cheese		Other	[1]
13	B	LCT-EF90	nd	CHI	76		Laboratory strain		Other	[4]
14	B	EnGen0029	1964	NOR	77		Fish burger		Other	[1]
15	B	EnGen0026	2000	DEU	296		Hospitalized patient		Clinical Isolate	[1]
16	Rec[a]	1_231_408	2005	USA	582	CC17	Hospitalized patient	Blood	Clinical Isolate	[2]
17	Rec	EnGen0002	2001	USA	117	CC17	Hospitalized patient	Feces	Hospital Surveillance	[1]
18	A1	EnGen0013	1997	ISR	80	CC17	Hospitalized patient	Blood	Clinical Isolate	[1]
20	A1	EnGen0034	2001	USA	117	CC17	Hospitalized patient	Urine	Clinical Isolate	[1]
21	A1	EnGen0046	2006	NLD	78	CC17	Hospitalized patient	Blood	Clinical Isolate	[1]
23	A1	E4452	2008	NLD	266		Dog	Feces	Animal	[3]
24	A1	EnGen0054	1999	ITA	78	CC17	Hospitalized patient	Catheter	Clinical Isolate	[1]
26	A1	EnGen0049	2010	PRT	78	CC17	Hospitalized patient		Hospital Surveillance	[1]
27	A1	EnGen0045	2010	LVA	78	CC17	Hospitalized patient		Hospital Surveillance	[1]
28	A1	EnGen0016	2000	GBR	64	CC17	Hospitalized patient		Hospital Unknown	[1]
29	A1	EnGen0036	2002	TZA	18	CC17	Hospitalized patient	Blood	Clinical Isolate	[1]
30	A1	EnGen0030	2002	NLD	325		Hospitalized patient	Blood	Clinical Isolate	[1]
31	A1	E4453	2008	NLD	192	CC17	Dog	Feces	Animal	[3]
33	A1	EnGen0050	2005	HUN	78	CC17	Hospitalized patient	Wound	Clinical Isolate	[1]

35	A1	Aus0004	1998	AUS	17	CC17	Hospitalized patient	Blood	Clinical Isolate	(17)
36	A1	C68	1996	USA	16	CC17	Hospitalized patient	Feces	Hospital Outbreak	Unpublished[d]
37	A1	E1162	1997	FRA	17	CC17	Hospitalized patient	Blood	Clinical Isolate	[3]
38	A1	EnGen0057	2008	DNK	78	CC17	Dog	Feces	Animal	[1]
39	A2	EnGen0018	2001	ZAF	159		Ostrich	Caecum	Animal	[1]
40	A2	EnGen0031	1960	NLD	22		Hospitalized patient	Pus	Clinical Isolate	[1]
41	A2	EnGen0007	2001	DEU	160		Mini pig	Feces	Animal	[1]
42	A2	EnGen0017	1998	NLD	92		Non-hospitalized individual	Feces	Non-hospitalized individual	[1]
43	A2	EnGen0025	1965	NLD	92		Hospitalized patient	Stomach	Clinical Isolate	[1]
44	A2	EnGen0009	1994	BEL	21		Bison	Rumen	Animal	[1]
45	A2	E1071	2000	NLD	32		Hospitalized patient	Feces	Hospital Surveillance	[3]
46	A2	EnGen0032	1959	NLD	104		Mouse		Animal	[1]
47	A2	D344SRF	nd	USA	25		Laboratory strain	Lab strain	Other	Unpublished[d]
48	A2	TC6	nd	USA	25		Laboratory strain	Lab strain	Other	Unpublished[d]
49	A2	EnGen0011	1998	FRA	26		Hospitalized patient	Blood	Clinical Isolate	[1]
50	A2	E1636	1961	NLD	106		Hospitalized patient	Blood	Clinical Isolate	[3]
51	A2	EnGen0010	1996	NLD	26		Turkey	Feces	Animal	[1]
52	A2	EnGen0048	2004	SWE	310		Chicken		Animal	[1]
53	A2	EnGen0005	1992	GBR	9		Chicken	Feces	Animal	[1]
54	A2	EnGen0022	1996	NLD	9		Turkey	Feces	Animal	[1]
55	A2	EnGen0043	2004	NLD	12		Chicken		Animal	[1]
56	A2	EnGen0027	1957	NLD	67		Hospitalized patient	Blood	Clinical Isolate	[1]
57	A2	EnGen0001	1995	BEL	158		Chicken		Animal	[1]
58	A2	E1679	1998	BRA	114		Hospitalized patient	Vascular catheter tip	Hospital Outbreak	[3]
59	A2	EnGen0024	2001	NLD	210		Hospitalized patient	Urine	Clinical Isolate	[1]
60	A2	EnGen0020	1995	BEL	27		Dog		Animal	[1]
61	A2	EnGen0012	1995	NLD	27		Hospitalized patient	Ascites	Clinical Isolate	[1]
62	A2	EnGen0044	2001	DNK	27		Poultry		Animal	[1]
64	A2	EnGen0004	1998	ESP	127		Hospitalized patient	Blood	Clinical Isolate	[1]
65	A2	EnGen0052	2002	NLD	332		Hospitalized patient	Blood	Clinical Isolate	[1]
66	A2	EnGen0039	1981	NLD	69		River water		Other	[1]
67	A2	EnGen0019	1995	DEU	151		Pig		Animal	[1]
68	A2	EnGen0040	1982	NLD	66		River water		Other	[1]
69	A2	EnGen0008	1995	ESP	5		Pig		Animal	[1]
70	A2	EnGen0021	2002	NLD	5		Hospitalized patient	Feces	Hospital Surveillance	[1]

71	A2	E1039	1998	NLD	42	Non-hospitalized individual	Feces	Non-hospitalized individual	[3]
72	A2	EnGen0014	1995	BEL	150	Pig		Animal	[1]
73	A2	EnGen0035	1979	NLD	66	Hospitalized patient	Gut	Clinical Isolate	[1]

[a] Rec stands for recombined
[b] Sequence-type determined by MLST
[c] CC stands for clonal complex
[d] released by the Broad Institute "Enterococcus Colonization Project".

[1] Lebreton F, van Schaik W, Manson McGuire A, Godfrey P, Griggs A, Mazumdar V, Corander J, Cheng L, Saif S, Young S, Zeng Q, Wortman J, Birren B, Willems RJL, Earl AM, Gilmore MS.2013. Emergence of epidemic multidrug-resistant Enterococcus faecium from animal and commensal strains. mBio 4:e00534-13. doi:10.1128/mBio.00534-13.

[2] Palmer KL, Godfrey P, Griggs A, Kos VN, Zucker J, Desjardins C, Cerqueira G, Gevers D, Walker S, Wortman J, Feldgarden M, Haas B, Birren B, and Gilmore MS. 2012. Comparative genomics of enterococci: variation in Enterococcus faecalis, clade structure in E. faecium, and defining characteristics of E. gallinarum and E. casseliflavus. mBio 3(1):e00318-11.

[3] van Schaik W, Top J, Riley DR, Boekhorst J, Vrijenhoek JEP, Schapendonk CME, Hendrickx APA, Nijman IJ, Bonten MJM, Tettelin H, and Willems RJL. 2010. Pyrosequencing-based comparative genome analysis of the nosocomial pathogen Enterococcus faecium and identification of a large transferable pathogenicity island. BMC Genomics 11:239.

[4] Chang D, Zhu Y, Zou Y, Fang X, Li T, Wang J, Guo Y, Su L, Xia J, Yang R, Fang C, and Liu C. 2012. Draft genome sequence of Enterococcus faecium strain LCT-EF90. J. Bacteriol. 194:3556–3557.
